# Supplementary material for: A MacMahon analysis view of cylindric partitions
Source: Ramanujan J. 2025 Sep 22;68(3):71. doi: 10.1007/s11139-025-01225-0 (PMC12454573; doi:10.1007/s11139-025-01225-0)
Supplement: Supplementary file 1 — (pdf 2709 KB) [file 11139_2025_1225_MOESM1_ESM.pdf]

In[1]:= << RISC`qMultiSum`

qMultiSum Package version 2.54
written by Axel Riese
Copyright Research Institute for Symbolic Computation (RISC),
Johannes Kepler University, Linz, Austria

In[2]:= << qFunctions.m

In order to use the full functionality you have to load the HolonomicFunctions package.

qFunctions by Jakob Ablinger and Ali K. Uncu – RISC – Version 1.1 (03 / 11 / 23) Help

```
In[3]:= (* qBinomial' from the paper *)
q1Binomial[a_, b_, q_] := If[a < 0 && b == 0, 1, qBinomial[a, b, q]];
```

Multisums and Bailey’s Theorem Applications

Common Definitions for the section

```
In[4]:= sumCount[k_, i_] := Sum[n[j]^2, {j, k - 1}] + Sum[n[j], {j, i, k - 1}];
sumCount[3, 2]
```

Out[5]= $n[1]^2 + n[2] + n[2]^2$

```
In[6]:= summationBounds[N_, k_] := If[k ≠ 1, Table[{n[i], 0, N}, {i, k - 1}], {{n[1], 0, 0}}];
summationBounds[5, 3]
```

Out[7]= {{n[1], 0, 5}, {n[2], 0, 5}}

```
In[8]:= MySum[a_, ind_List] := Module[{s}, s[a, Sequence @@ ind] /. s → Summ];
MySum[a[n[1], n[2]], summationBounds[5, 3]]
```

Out[9]= Summ[a[n[1], n[2]], {n[1], 0, 5}, {n[2], 0, 5}]

The Foda-Quano Andrews-Gordon Sums

Theorem 1.2 in paper (Recall k>=2)

In[10]:=
```
FQAndrewsGordonSumLHS[N_, k_, i_] :=
  MySum[q^sumCount[k, i] Product[qBinomial[2 N - 2 Sum[n[jj], {jj, j - 1}] - n[j] -
        n[j + 1] - Max[j - i + 1, 0], n[j] - n[j + 1], q], {j, k - 2}] × qBinomial[
      2 N - 2 Sum[n[jj], {jj, k - 1}] + n[k - 1] - k + i, n[k - 1], q], summationBounds[N, k]];
FQAndrewsGordonSumLHS[n, 3, 2] /. Summ → Sum
```

Out[11]=

$$\sum_{n[1]=0}^{n}\sum_{n[2]=0}^{n} q^{n[1]^2+n[2]+n[2]^2}\, \text{qBinomial}[2n-n[1]-n[2],\, n[1]-n[2],\, q] \times \text{qBinomial}[-1+2n+n[2]-2(n[1]+n[2]),\, n[2],\, q]$$

In[12]:=
```
FQAndrewsGordonSumRHS[N_, k_, i_] := Sum[(-1)^r q^(r ((2 k + 1) r + 2 k - 2 i + 1) / 2)
    qBinomial[2 N, N - Floor[(i - k - (2 k + 1) r) / 2], q] , {r, -N, N}];
FQAndrewsGordonSumRHS[n, 3, 2]
```

Out[13]=

$$\sum_{r=-n}^{n} (-1)^r q^{\frac{1}{2} r (3+7r)}\, \text{qBinomial}\left[2n,\, n-\text{Floor}\left[\frac{1}{2}(-1-7r)\right],\, q\right]$$

In[14]:=
```
(* Show that initial conditions match for 10≥N≥0, 4≥k≥2, and k≥i≥1  *)
Table[Expand[(FQAndrewsGordonSumLHS[N, k, i] /. Summ → Sum) -
   FQAndrewsGordonSumRHS[N, k, i]], {N, 0, 10}, {k, 2, 4}, {i, k}]
```

Out[14]=
```
{{{0, 0}, {0, 0, 0}, {0, 0, 0, 0}},
 {{0, 0}, {0, 0, 0}, {0, 0, 0, 0}}, {{0, 0}, {0, 0, 0}, {0, 0, 0, 0}},
 {{0, 0}, {0, 0, 0}, {0, 0, 0, 0}}, {{0, 0}, {0, 0, 0}, {0, 0, 0, 0}},
 {{0, 0}, {0, 0, 0}, {0, 0, 0, 0}}, {{0, 0}, {0, 0, 0}, {0, 0, 0, 0}},
 {{0, 0}, {0, 0, 0}, {0, 0, 0, 0}}, {{0, 0}, {0, 0, 0}, {0, 0, 0, 0}},
 {{0, 0}, {0, 0, 0}, {0, 0, 0, 0}}, {{0, 0}, {0, 0, 0}, {0, 0, 0, 0}}}
```

Berkovich-Uncu Bailey Theorem Application (Theorem 8.2)

In[15]:=
```
BUBaileyOfFQAndrewsGordonSumRHS[N_, p_, k_, i_] :=
  Sum[(-1)^r q^(r ((2 k + 1) r + 2 k - 2 i + 1) / 2 + p Floor[(i - k - (2 k + 1) r) / 2]^2)
    qBinomial[2 N, N - Floor[(i - k - (2 k + 1) r) / 2], q] , {r, -N, N}];
BUBaileyOfFQAndrewsGordonSumRHS[5, 3, 5, 2] // Expand
```

Out[16]=

$$q^{12}+q^{13}+2q^{14}+3q^{15}+4q^{16}+5q^{17}+7q^{18}+8q^{19}+9q^{20}+10q^{21}+10q^{22}+10q^{23}+10q^{24}+9q^{25}+8q^{26}+7q^{27}+5q^{28}+4q^{29}+3q^{30}+2q^{31}+q^{32}+q^{33}-q^{50}-q^{51}-q^{52}-q^{53}-q^{54}-q^{55}-q^{56}-q^{57}-q^{58}-q^{59}$$

In[17]:=
```
BUBaileyFactor[L_, r_] :=
 q^(r^2) qPochhammer[q, q, 2 L] / qPochhammer[q, q, L - r] / qPochhammer[q, q, 2 r]
```

In[18]:=
```
summationBoundsM[N_, p_] :=
  If[p ≠ 1, Table[{m[i], 0, m[i + 1]}, {i, p, 1, -1}], {{m[1], 0, N}}] /. m[p + 1] → N;
```

In[19]:=
```
summationBoundsM[N, 2]
```

Out[19]=
```
{{m[2], 0, N}, {m[1], 0, m[2]}}
```

In[20]:=
```
BUBaileyOfFQAndrewsGordonSumLHS[N_, p_, k_, i_] := Module[{f},
  If[ p == 1,
   f = BUBaileyFactor[N, m[1]] × FQAndrewsGordonSumLHS[m[1], k, i];
   ,
   f = FQAndrewsGordonSumLHS[m[1], k, i];
   Do[f = BUBaileyFactor[m[j + 1], m[j]] f, {j, 1, p}];
  ];
  MySum[f /. m[p + 1] → N, summationBoundsM[N, p]] /. Summ → Sum
 ]
```

In[21]:=
```
(* Show that initial conditions match for 5≥N≥0, 5≥p≥1, 5≥k≥2, and k≥i≥1  *)
Table[BUBaileyOfFQAndrewsGordonSumLHS[NN, pp, kk, ii] -
   BUBaileyOfFQAndrewsGordonSumRHS[NN, pp, kk, ii] ,
  {NN, 0, 5}, {pp, 5}, {kk, 2, 4}, {ii, kk}] // Simplify
```

Out[21]=
```
{{{{0, 0}, {0, 0, 0}, {0, 0, 0, 0}}, {{0, 0}, {0, 0, 0}, {0, 0, 0, 0}},
  {{0, 0}, {0, 0, 0}, {0, 0, 0, 0}}, {{0, 0}, {0, 0, 0}, {0, 0, 0, 0}},
  {{0, 0}, {0, 0, 0}, {0, 0, 0, 0}}}, {{{0, 0}, {0, 0, 0}, {0, 0, 0, 0}},
  {{0, 0}, {0, 0, 0}, {0, 0, 0, 0}}, {{0, 0}, {0, 0, 0}, {0, 0, 0, 0}},
  {{0, 0}, {0, 0, 0}, {0, 0, 0, 0}}, {{0, 0}, {0, 0, 0}, {0, 0, 0, 0}}},
 {{{0, 0}, {0, 0, 0}, {0, 0, 0, 0}}, {{0, 0}, {0, 0, 0}, {0, 0, 0, 0}},
  {{0, 0}, {0, 0, 0}, {0, 0, 0, 0}}, {{0, 0}, {0, 0, 0}, {0, 0, 0, 0}},
  {{0, 0}, {0, 0, 0}, {0, 0, 0, 0}}}, {{{0, 0}, {0, 0, 0}, {0, 0, 0, 0}},
  {{0, 0}, {0, 0, 0}, {0, 0, 0, 0}}, {{0, 0}, {0, 0, 0}, {0, 0, 0, 0}},
  {{0, 0}, {0, 0, 0}, {0, 0, 0, 0}}, {{0, 0}, {0, 0, 0}, {0, 0, 0, 0}}},
 {{{0, 0}, {0, 0, 0}, {0, 0, 0, 0}}, {{0, 0}, {0, 0, 0}, {0, 0, 0, 0}},
  {{0, 0}, {0, 0, 0}, {0, 0, 0, 0}}, {{0, 0}, {0, 0, 0}, {0, 0, 0, 0}},
  {{0, 0}, {0, 0, 0}, {0, 0, 0, 0}}}, {{{0, 0}, {0, 0, 0}, {0, 0, 0, 0}},
  {{0, 0}, {0, 0, 0}, {0, 0, 0, 0}}, {{0, 0}, {0, 0, 0}, {0, 0, 0, 0}},
  {{0, 0}, {0, 0, 0}, {0, 0, 0, 0}}, {{0, 0}, {0, 0, 0}, {0, 0, 0, 0}}}}
```

The Conjectural Andrews-Gordon Sums

Conjecture 1.3 (Recall k>=2)

In[22]:=
```
LUAndrewsGordonSumLHS[N_, k_, i_] :=
  MySum[q^sumCount[k, i] Product[q1Binomial[2 N - 2 Sum[n[jj], {jj, j - 1}] -
       n[j] - n[j + 1] - 2 Max[j - i + 1, 0], n[j] - n[j + 1], q], {j, k - 2}] ×
    q1Binomial[2 N - 2 Sum[n[jj], {jj, k - 1}] + n[k - 1] - 2 k + 2 i, n[k - 1], q],
   summationBounds[N, k]];
```

In[23]:=
```
LUAndrewsGordonSumRHS[N_, k_, i_] := Sum[(-1)^r q^(r ((2 k + 1) r + 2 k - 2 i + 1) / 2)
    qBinomial[2 N, N - (2 k + 1) / 2 r + (2 k - 2 i + 1) ((-1)^r - 1) / 4, q] , {r, -N, N}];
```

In[24]:=
```
(* Show that initial conditions match for 10≥N≥0, 4≥k≥2, and k≥i≥1  *)
Table[Expand[(LUAndrewsGordonSumLHS[N, k, i] /. Summ → Sum) -
   LUAndrewsGordonSumRHS[N, k, i]], {N, 0, 10}, {k, 2, 4}, {i, k}]
```

Out[24]=
```
{{{0, 0}, {0, 0, 0}, {0, 0, 0, 0}},
 {{0, 0}, {0, 0, 0}, {0, 0, 0, 0}}, {{0, 0}, {0, 0, 0}, {0, 0, 0, 0}},
 {{0, 0}, {0, 0, 0}, {0, 0, 0, 0}}, {{0, 0}, {0, 0, 0}, {0, 0, 0, 0}},
 {{0, 0}, {0, 0, 0}, {0, 0, 0, 0}}, {{0, 0}, {0, 0, 0}, {0, 0, 0, 0}},
 {{0, 0}, {0, 0, 0}, {0, 0, 0, 0}}, {{0, 0}, {0, 0, 0}, {0, 0, 0, 0}},
 {{0, 0}, {0, 0, 0}, {0, 0, 0, 0}}, {{0, 0}, {0, 0, 0}, {0, 0, 0, 0}}}
```

Berkovich-Uncu Bailey Theorem Application (Conjecture 8.4)

In[25]:=
```
BUBaileyOfLUAndrewsGordonSumRHS[N_, p_, k_, i_] :=
  Sum[(-1)^r q^(r ((2 k + 1) r + 2 k - 2 i + 1) / 2 + p ×
        ((2 k + 1) / 2 r - (2 k - 2 i + 1) ((-1)^r - 1) / 4)^2)
    qBinomial[2 N, N - (2 k + 1) / 2 r + (2 k - 2 i + 1) ((-1)^r - 1) / 4, q] , {r, -N, N}];
BUBaileyOfLUAndrewsGordonSumRHS[5, 3, 5, 2] // Expand
```

Out[26]=

$$1+q+2q^{2}+3q^{3}+5q^{4}+7q^{5}+9q^{6}+11q^{7}+14q^{8}+16q^{9}+18q^{10}+19q^{11}+20q^{12}+20q^{13}+18q^{14}+17q^{15}+14q^{16}+11q^{17}+7q^{18}+4q^{19}-3q^{21}-6q^{22}-8q^{23}-9q^{24}-9q^{25}-10q^{26}-9q^{27}-8q^{28}-7q^{29}-5q^{30}-4q^{31}-3q^{32}-2q^{33}-q^{34}-q^{35}$$

In[27]:=
```
BUBaileyFactor[L_, r_] :=
 q^(r^2) qPochhammer[q, q, 2 L] / qPochhammer[q, q, L - r] / qPochhammer[q, q, 2 r]
```

In[28]:=
```
summationBoundsM[N_, p_] :=
  If[p ≠ 1, Table[{m[i], 0, m[i + 1]}, {i, p, 1, -1}], {{m[1], 0, N}}] /. m[p + 1] → N;
summationBoundsM[N, 2]
```

Out[29]=
```
{{m[2], 0, N}, {m[1], 0, m[2]}}
```

In[30]:=
```
BUBaileyOfLUAndrewsGordonSumLHS[NN_, p_, k_, i_] := Module[{f},
  If[ p == 1,
   f = BUBaileyFactor[NN, m[1]] × LUAndrewsGordonSumLHS[m[1], k, i];
   ,
   f = LUAndrewsGordonSumLHS[m[1], k, i];
   Do[f = BUBaileyFactor[m[j + 1], m[j]] f, {j, 1, p}];
  ];
  MySum[f /. m[p + 1] → NN, summationBoundsM[NN, p]] /. Summ → Sum
 ]
```

In[31]:=
```
(* Show that initial conditions match for 5≥N≥1, 5≥p≥1, 5≥k≥2,
and k≥i≥1  *)Table[BUBaileyOfLUAndrewsGordonSumLHS[NN, pp, kk, ii] -
   BUBaileyOfLUAndrewsGordonSumRHS[NN, pp, kk, ii],
  {NN, 0, 5}, {pp, 5}, {kk, 2, 5}, {ii, kk}] // Simplify
```

Out[31]=
```
{{{{0, 0}, {0, 0, 0}, {0, 0, 0, 0}, {0, 0, 0, 0, 0}},
  {{0, 0}, {0, 0, 0}, {0, 0, 0, 0}, {0, 0, 0, 0, 0}},
  {{0, 0}, {0, 0, 0}, {0, 0, 0, 0}, {0, 0, 0, 0, 0}},
  {{0, 0}, {0, 0, 0}, {0, 0, 0, 0}, {0, 0, 0, 0, 0}},
  {{0, 0}, {0, 0, 0}, {0, 0, 0, 0}, {0, 0, 0, 0, 0}}},
 {{{0, 0}, {0, 0, 0}, {0, 0, 0, 0}, {0, 0, 0, 0, 0}},
  {{0, 0}, {0, 0, 0}, {0, 0, 0, 0}, {0, 0, 0, 0, 0}},
  {{0, 0}, {0, 0, 0}, {0, 0, 0, 0}, {0, 0, 0, 0, 0}},
  {{0, 0}, {0, 0, 0}, {0, 0, 0, 0}, {0, 0, 0, 0, 0}},
  {{0, 0}, {0, 0, 0}, {0, 0, 0, 0}, {0, 0, 0, 0, 0}}},
 {{{0, 0}, {0, 0, 0}, {0, 0, 0, 0}, {0, 0, 0, 0, 0}},
  {{0, 0}, {0, 0, 0}, {0, 0, 0, 0}, {0, 0, 0, 0, 0}},
  {{0, 0}, {0, 0, 0}, {0, 0, 0, 0}, {0, 0, 0, 0, 0}},
  {{0, 0}, {0, 0, 0}, {0, 0, 0, 0}, {0, 0, 0, 0, 0}},
  {{0, 0}, {0, 0, 0}, {0, 0, 0, 0}, {0, 0, 0, 0, 0}}},
 {{{0, 0}, {0, 0, 0}, {0, 0, 0, 0}, {0, 0, 0, 0, 0}},
  {{0, 0}, {0, 0, 0}, {0, 0, 0, 0}, {0, 0, 0, 0, 0}},
  {{0, 0}, {0, 0, 0}, {0, 0, 0, 0}, {0, 0, 0, 0, 0}},
  {{0, 0}, {0, 0, 0}, {0, 0, 0, 0}, {0, 0, 0, 0, 0}},
  {{0, 0}, {0, 0, 0}, {0, 0, 0, 0}, {0, 0, 0, 0, 0}}},
 {{{0, 0}, {0, 0, 0}, {0, 0, 0, 0}, {0, 0, 0, 0, 0}},
  {{0, 0}, {0, 0, 0}, {0, 0, 0, 0}, {0, 0, 0, 0, 0}},
  {{0, 0}, {0, 0, 0}, {0, 0, 0, 0}, {0, 0, 0, 0, 0}},
  {{0, 0}, {0, 0, 0}, {0, 0, 0, 0}, {0, 0, 0, 0, 0}},
  {{0, 0}, {0, 0, 0}, {0, 0, 0, 0}, {0, 0, 0, 0, 0}}},
 {{{0, 0}, {0, 0, 0}, {0, 0, 0, 0}, {0, 0, 0, 0, 0}},
  {{0, 0}, {0, 0, 0}, {0, 0, 0, 0}, {0, 0, 0, 0, 0}},
  {{0, 0}, {0, 0, 0}, {0, 0, 0, 0}, {0, 0, 0, 0, 0}},
  {{0, 0}, {0, 0, 0}, {0, 0, 0, 0}, {0, 0, 0, 0, 0}},
  {{0, 0}, {0, 0, 0}, {0, 0, 0, 0}, {0, 0, 0, 0, 0}}}}
```

The Foda-Quano Bressoud Sums

Theorem 1.5 (Recall k>=2)

In[32]:=
```
FQBressoudSumLHS[N_, k_, i_] :=
  MySum[q^sumCount[k, i] Product[qBinomial[2 N - 2 Sum[n[jj], {jj, j - 1}] -
       n[j] - n[j + 1] + Min[k - i, k - j - 1], n[j] - n[j + 1], q], {j, k - 2}] ×
     qBinomial[N - Sum[n[jj], {jj, k - 2}], n[k - 1], q^2], summationBounds[N, k]];
FQBressoudSumLHS[n, 5, 2] /. Summ → Sum
```

Out[33]=

$$\sum_{n[1]=0}^{n}\sum_{n[2]=0}^{n}\sum_{n[3]=0}^{n}\sum_{n[4]=0}^{n} q^{n[1]^2+n[2]+n[2]^2+n[3]+n[3]^2+n[4]+n[4]^2}\,\text{qBinomial}[3+2n-n[1]-n[2],\ n[1]-n[2],q]\times\text{qBinomial}[2+2n-2n[1]-n[2]-n[3],\ n[2]-n[3],q]\times\text{qBinomial}\left[n-n[1]-n[2]-n[3],\ n[4],\ q^2\right]\times\text{qBinomial}[1+2n-2(n[1]+n[2])-n[3]-n[4],\ n[3]-n[4],q]$$

In[34]:=
```
FQBressoudSumRHS[N_, k_, i_] :=
  Sum[(-1)^r q^(r (k r + k - i)) qBinomial[2 N + k - i, N - k r, q] , {r, -N, N}];
FQBressoudSumRHS[n, 5, 2]
```

Out[35]=

$$\sum_{r=-n}^{n} (-1)^r q^{r\,(3+5r)}\,\text{qBinomial}[3+2n,\ n-5r,\ q]$$

In[36]:=
```
(* Show that initial conditions match for 10≥N≥0, 4≥k≥2, and k≥i≥1  *)
Table[Expand[(FQBressoudSumLHS[N, k, i] /. Summ → Sum) - FQBressoudSumRHS[N, k, i]],
 {N, 0, 10}, {k, 2, 4}, {i, k}]
```

Out[36]=

```
{{{0, 0}, {0, 0, 0}, {0, 0, 0, 0}},
 {{0, 0}, {0, 0, 0}, {0, 0, 0, 0}}, {{0, 0}, {0, 0, 0}, {0, 0, 0, 0}},
 {{0, 0}, {0, 0, 0}, {0, 0, 0, 0}}, {{0, 0}, {0, 0, 0}, {0, 0, 0, 0}},
 {{0, 0}, {0, 0, 0}, {0, 0, 0, 0}}, {{0, 0}, {0, 0, 0}, {0, 0, 0, 0}},
 {{0, 0}, {0, 0, 0}, {0, 0, 0, 0}}, {{0, 0}, {0, 0, 0}, {0, 0, 0, 0}},
 {{0, 0}, {0, 0, 0}, {0, 0, 0, 0}}, {{0, 0}, {0, 0, 0}, {0, 0, 0, 0}}}
```

Berkovich-Uncu Bailey Theorem Application (only applicable when i=k and i=k-1 - Theorem 8.7)

In[37]:=
```
BUBaileyOfFQBressoudSumRHS[N_, p_, k_, i_, A_] := Sum[(-1)^r
    q^(r (k r + k - i) + p (k r)^2 + A p (k r)) qBinomial[2 N + k - i, N - k r, q] , {r, -N, N}];
BUBaileyOfFQBressoudSumRHS[5, 3, 5, 4, 1] // Expand
```

Out[38]=

$$1+q+2q^2+3q^3+5q^4+7q^5+10q^6+12q^7+16q^8+19q^9+23q^{10}+25q^{11}+29q^{12}+30q^{13}+32q^{14}+32q^{15}+32q^{16}+30q^{17}+29q^{18}+25q^{19}+23q^{20}+19q^{21}+16q^{22}+12q^{23}+10q^{24}+7q^{25}+5q^{26}+3q^{27}+2q^{28}+q^{29}+q^{30}-q^{64}-q^{65}-q^{66}-q^{67}-q^{68}-q^{69}-q^{70}-q^{71}-q^{72}-q^{73}-q^{74}-q^{96}$$

In[39]:=
```
BUBaileyFactor[L_, r_, A_] := q^(r^2 + A r)
  qPochhammer[q, q, 2 L + A] / qPochhammer[q, q, L - r] / qPochhammer[q, q, 2 r + A]
```

In[40]:=
```
summationBoundsM[N_, p_] :=
  If[p ≠ 1, Table[{m[i], 0, m[i + 1]}, {i, p, 1, -1}], {{m[1], 0, N}}] /. m[p + 1] → N;
```

In[41]:=
```
summationBoundsM[N, 2]
```
Out[41]=
```
{{m[2], 0, N}, {m[1], 0, m[2]}}
```
In[42]:=
```
BUBaileyOfFQBressoudSumLHS[N_, p_, k_, i_] := Module[{f},
  If[ p == 1,
   f = BUBaileyFactor[N, m[1], k - i] × FQBressoudSumLHS[m[1], k, i];
   ,
   f = FQBressoudSumLHS[m[1], k, i];
   Do[f = BUBaileyFactor[m[j + 1], m[j], k - i] f, {j, 1, p}];
  ];
  MySum[f /. m[p + 1] → N, summationBoundsM[N, p]] /. Summ → Sum
 ]
```
In[43]:=
```
(* Show that initial conditions match for 5≥N≥0, 5≥p≥1, 5≥k≥2,
and k≥i≥1  *)Table[BUBaileyOfFQBressoudSumLHS[NN, pp, kk, ii] -
   BUBaileyOfFQBressoudSumRHS[NN, pp, kk, ii, kk - ii],
  {NN, 0, 5}, {pp, 5}, {kk, 2, 5}, {ii, kk - 1, kk}] // Simplify
```
Out[43]=
```
{{{{0, 0}, {0, 0}, {0, 0}, {0, 0}}, {{0, 0}, {0, 0}, {0, 0}, {0, 0}},
  {{0, 0}, {0, 0}, {0, 0}, {0, 0}}, {{0, 0}, {0, 0}, {0, 0}, {0, 0}},
  {{0, 0}, {0, 0}, {0, 0}, {0, 0}}}, {{{0, 0}, {0, 0}, {0, 0}, {0, 0}},
  {{0, 0}, {0, 0}, {0, 0}, {0, 0}}, {{0, 0}, {0, 0}, {0, 0}, {0, 0}},
  {{0, 0}, {0, 0}, {0, 0}, {0, 0}}, {{0, 0}, {0, 0}, {0, 0}, {0, 0}}},
 {{{0, 0}, {0, 0}, {0, 0}, {0, 0}}, {{0, 0}, {0, 0}, {0, 0}, {0, 0}},
  {{0, 0}, {0, 0}, {0, 0}, {0, 0}}, {{0, 0}, {0, 0}, {0, 0}, {0, 0}},
  {{0, 0}, {0, 0}, {0, 0}, {0, 0}}}, {{{0, 0}, {0, 0}, {0, 0}, {0, 0}},
  {{0, 0}, {0, 0}, {0, 0}, {0, 0}}, {{0, 0}, {0, 0}, {0, 0}, {0, 0}},
  {{0, 0}, {0, 0}, {0, 0}, {0, 0}}, {{0, 0}, {0, 0}, {0, 0}, {0, 0}}},
 {{{0, 0}, {0, 0}, {0, 0}, {0, 0}}, {{0, 0}, {0, 0}, {0, 0}, {0, 0}},
  {{0, 0}, {0, 0}, {0, 0}, {0, 0}}, {{0, 0}, {0, 0}, {0, 0}, {0, 0}},
  {{0, 0}, {0, 0}, {0, 0}, {0, 0}}}, {{{0, 0}, {0, 0}, {0, 0}, {0, 0}},
  {{0, 0}, {0, 0}, {0, 0}, {0, 0}}, {{0, 0}, {0, 0}, {0, 0}, {0, 0}},
  {{0, 0}, {0, 0}, {0, 0}, {0, 0}}, {{0, 0}, {0, 0}, {0, 0}, {0, 0}}}}
```

The Conjectural Bressoud Sums

Conjecture 1.6   (Recall k>=2)

In[44]:=
```
LUBressoudSumLHS[N_, k_, i_] :=
  MySum[q^sumCount[k, i] Product[q1Binomial[2 N - 2 Sum[n[jj], {jj, j - 1}] -
       n[j] - n[j + 1] - 2 Max[j - i + 1, 0], n[j] - n[j + 1], q], {j, k - 2}] ×
    q1Binomial[N - Sum[n[jj], {jj, k - 2}] - k + i, n[k - 1], q^2], summationBounds[N, k]];
```
In[45]:=
```
LUBressodSumRHS[N_, k_, i_] := Sum[(-1)^r q^( r (k r + k - i) )
    qBinomial[2 N, N - k r + (k - i) ((-1)^r - 1) / 2, q] , {r, -N, N}];
```

In[46]:=
```
(* Show that initial conditions match for 10≥N≥0, 4≥k≥2, and k≥i≥1  *)
Table[Expand[(LUBressoudSumLHS[N, k, i] /. {Summ → Sum}) - LUBressodSumRHS[N, k, i]],
 {N, 0, 10}, {k, 2, 4}, {i, k}]
```
Out[46]=
```
{{{0, 0}, {0, 0, 0}, {0, 0, 0, 0}},
 {{0, 0}, {0, 0, 0}, {0, 0, 0, 0}}, {{0, 0}, {0, 0, 0}, {0, 0, 0, 0}},
 {{0, 0}, {0, 0, 0}, {0, 0, 0, 0}}, {{0, 0}, {0, 0, 0}, {0, 0, 0, 0}},
 {{0, 0}, {0, 0, 0}, {0, 0, 0, 0}}, {{0, 0}, {0, 0, 0}, {0, 0, 0, 0}},
 {{0, 0}, {0, 0, 0}, {0, 0, 0, 0}}, {{0, 0}, {0, 0, 0}, {0, 0, 0, 0}},
 {{0, 0}, {0, 0, 0}, {0, 0, 0, 0}}, {{0, 0}, {0, 0, 0}, {0, 0, 0, 0}}}
```

Berkovich-Uncu Bailey Theorem Application (Conjecture 8.8)

In[47]:=
```
BUBaileyOfLUBressoudSumRHS[N_, p_, k_, i_] :=
  Sum[(-1)^r q^( r (k r + k - i) + p (k r - (k - i) ((-1)^r - 1) / 2)^2 )
    qBinomial[2 N, N - k r + (k - i) ((-1)^r - 1) / 2, q] , {r, -N, N}];
BUBaileyOfLUBressoudSumRHS[5, 3, 5, 2] // Expand
```
Out[48]=

$$1+q+2q^2+3q^3+5q^4+7q^5+9q^6+11q^7+14q^8+16q^9+18q^{10}+19q^{11}+20q^{12}+20q^{13}+18q^{14}+17q^{15}+14q^{16}+11q^{17}+7q^{18}+4q^{19}-3q^{21}-6q^{22}-8q^{23}-9q^{24}-9q^{25}-10q^{26}-9q^{27}-8q^{28}-7q^{29}-5q^{30}-4q^{31}-3q^{32}-2q^{33}-q^{34}-q^{35}$$

In[49]:=
```
BUBaileyFactor[L_, r_] :=
 q^(r^2) qPochhammer[q, q, 2 L] / qPochhammer[q, q, L - r] / qPochhammer[q, q, 2 r]
```

In[50]:=
```
summationBoundsM[N_, p_] :=
  If[p ≠ 1, Table[{m[i], 0, m[i + 1]}, {i, p, 1, -1}], {{m[1], 0, N}}] /. m[p + 1] → N;
summationBoundsM[N, 2]
```
Out[51]=
```
{{m[2], 0, N}, {m[1], 0, m[2]}}
```

In[52]:=
```
BUBaileyOfLUBressoudSumLHS[NN_, p_, k_, i_] := Module[{f},
  If[ p == 1,
   f = BUBaileyFactor[NN, m[1]] × LUBressoudSumLHS[m[1], k, i];
   ,
   f = LUBressoudSumLHS[m[1], k, i];
   Do[f = BUBaileyFactor[m[j + 1], m[j]] f, {j, 1, p}];
  ];
  MySum[f /. m[p + 1] → NN, summationBoundsM[NN, p]] /. Summ → Sum
 ]
```

In[53]:=
```
(* Show that initial conditions match for 5≥N≥0, 5≥p≥1, 5≥k≥2,
and k≥i≥1  *)Table[BUBaileyOfLUBressoudSumLHS[NN, pp, kk, ii] -
   BUBaileyOfLUBressoudSumRHS[NN, pp, kk, ii],
  {NN, 0, 5}, {pp, 5}, {kk, 2, 5}, {ii, kk}] // Simplify
```

Out[53]=
```
{{{{0, 0}, {0, 0, 0}, {0, 0, 0, 0}, {0, 0, 0, 0, 0}},
  {{0, 0}, {0, 0, 0}, {0, 0, 0, 0}, {0, 0, 0, 0, 0}},
  {{0, 0}, {0, 0, 0}, {0, 0, 0, 0}, {0, 0, 0, 0, 0}},
  {{0, 0}, {0, 0, 0}, {0, 0, 0, 0}, {0, 0, 0, 0, 0}},
  {{0, 0}, {0, 0, 0}, {0, 0, 0, 0}, {0, 0, 0, 0, 0}}},
 {{{0, 0}, {0, 0, 0}, {0, 0, 0, 0}, {0, 0, 0, 0, 0}},
  {{0, 0}, {0, 0, 0}, {0, 0, 0, 0}, {0, 0, 0, 0, 0}},
  {{0, 0}, {0, 0, 0}, {0, 0, 0, 0}, {0, 0, 0, 0, 0}},
  {{0, 0}, {0, 0, 0}, {0, 0, 0, 0}, {0, 0, 0, 0, 0}},
  {{0, 0}, {0, 0, 0}, {0, 0, 0, 0}, {0, 0, 0, 0, 0}}},
 {{{0, 0}, {0, 0, 0}, {0, 0, 0, 0}, {0, 0, 0, 0, 0}},
  {{0, 0}, {0, 0, 0}, {0, 0, 0, 0}, {0, 0, 0, 0, 0}},
  {{0, 0}, {0, 0, 0}, {0, 0, 0, 0}, {0, 0, 0, 0, 0}},
  {{0, 0}, {0, 0, 0}, {0, 0, 0, 0}, {0, 0, 0, 0, 0}},
  {{0, 0}, {0, 0, 0}, {0, 0, 0, 0}, {0, 0, 0, 0, 0}}},
 {{{0, 0}, {0, 0, 0}, {0, 0, 0, 0}, {0, 0, 0, 0, 0}},
  {{0, 0}, {0, 0, 0}, {0, 0, 0, 0}, {0, 0, 0, 0, 0}},
  {{0, 0}, {0, 0, 0}, {0, 0, 0, 0}, {0, 0, 0, 0, 0}},
  {{0, 0}, {0, 0, 0}, {0, 0, 0, 0}, {0, 0, 0, 0, 0}},
  {{0, 0}, {0, 0, 0}, {0, 0, 0, 0}, {0, 0, 0, 0, 0}}},
 {{{0, 0}, {0, 0, 0}, {0, 0, 0, 0}, {0, 0, 0, 0, 0}},
  {{0, 0}, {0, 0, 0}, {0, 0, 0, 0}, {0, 0, 0, 0, 0}},
  {{0, 0}, {0, 0, 0}, {0, 0, 0, 0}, {0, 0, 0, 0, 0}},
  {{0, 0}, {0, 0, 0}, {0, 0, 0, 0}, {0, 0, 0, 0, 0}},
  {{0, 0}, {0, 0, 0}, {0, 0, 0, 0}, {0, 0, 0, 0, 0}}},
 {{{0, 0}, {0, 0, 0}, {0, 0, 0, 0}, {0, 0, 0, 0, 0}},
  {{0, 0}, {0, 0, 0}, {0, 0, 0, 0}, {0, 0, 0, 0, 0}},
  {{0, 0}, {0, 0, 0}, {0, 0, 0, 0}, {0, 0, 0, 0, 0}},
  {{0, 0}, {0, 0, 0}, {0, 0, 0, 0}, {0, 0, 0, 0, 0}},
  {{0, 0}, {0, 0, 0}, {0, 0, 0, 0}, {0, 0, 0, 0, 0}}}}
```

Proofs of The Identities

Proofs of Identities (3.1) and (3.2)

Proof or (3.1)

In[54]:=
```
(* Sums in the (3.1) identity*)
rec3p1LHSExp[n_] := Sum[q^(n1^2 + n1) q1Binomial[n - 1, n1, q^2], {n1, 0, n}];
rec3p1RHSExp[n_] :=
 Sum[(-1)^r q^(2 r^2 + r) qBinomial[2 n, n - 2 r + ((-1)^r - 1) / 2, q], {r, -n, n}]
```

In[56]:=
```
(* Checking that the initial values match *)
Table[Expand[rec3p1LHSExp[n] - rec3p1RHSExp[n]], {n, 0, 20}]
```

Out[56]=
```
{0, 0, 0, 0, 0, 0, 0, 0, 0, 0, 0, 0, 0, 0, 0, 0, 0, 0, 0, 0, 0}
```

In[57]:=
```
(* Recurrences of the sides of (3.1) *)
rec3p1LHS = qSR[qFR[q^(n1^2 + n1) qBinomial[n - 1, n1, q^2], {n}, {n1}, {0}, {1}]]
```

Out[57]=

$$\left\{\left(1+q^{2n}\right)\mathrm{SUM}[n]-\mathrm{SUM}[1+n]==0\right\}$$

In[58]:=
```
(* The RHS sum of (3.1) needs to be split into
 two (r→ 2r and r→ 2r+1) before finding recurrences. *)
rec3p1RHSsumSplit1 =
 qSR[qFR[((-1)^r q^(2 r^2 + r) qBinomial[2 n, n - 2 r + ((-1)^r - 1) / 2, q]) /. r → 2 r /.
    (-1)^(2 r) → 1, {n}, {r}, {0}, {2}]]
```

Out[58]=

$$\left\{-q^{6}\left(-1+q^{n}\right)\left(1+q^{n}\right)\left(1+q^{2n}\right)\left(-1+q^{1+n}\right)\left(1+q^{1+n}\right)\left(1+q^{2+2n}\right)\mathrm{SUM}[n]+ q^{3}(1+q)\left(1+q^{2}\right)\left(1+q^{2+2n}\right)\left(1+q^{4+4n}\right)\mathrm{SUM}[1+n]- q\left(1+q^{2}\right)\left(1+q+q^{2}+q^{3+2n}+2q^{4+2n}+q^{5+2n}+q^{6+4n}+q^{7+4n}+q^{8+4n}\right)\mathrm{SUM}[2+n]+ (1+q)\left(1+q^{2}\right)\left(1+q^{4+2n}\right)\mathrm{SUM}[3+n]-\mathrm{SUM}[4+n]==0\right\}$$

In[59]:=
```
rec3p1RHSsumSplit2 =
 qSR[qFR[((-1)^r q^(2 r^2 + r) qBinomial[2 n, n - 2 r + ((-1)^r - 1) / 2, q]) /. r → 2 r + 1 /.
    (-1)^(2 r + 1) → -1, {n}, {r}, {0}, {2}]]
```

Out[59]=

$$\left\{-q^{6}\left(-1+q^{n}\right)\left(1+q^{n}\right)\left(1+q^{2n}\right)\left(-1+q^{1+n}\right)\left(1+q^{1+n}\right)\left(1+q^{2+2n}\right)\mathrm{SUM}[n]+ q^{3}(1+q)\left(1+q^{2}\right)\left(1+q^{2+2n}\right)\left(1+q^{4+4n}\right)\mathrm{SUM}[1+n]- q\left(1+q^{2}\right)\left(1+q+q^{2}+q^{3+2n}+2q^{4+2n}+q^{5+2n}+q^{6+4n}+q^{7+4n}+q^{8+4n}\right)\mathrm{SUM}[2+n]+ (1+q)\left(1+q^{2}\right)\left(1+q^{4+2n}\right)\mathrm{SUM}[3+n]-\mathrm{SUM}[4+n]==0\right\}$$

In[60]:=
```
rec3p1RHSsumSplit1 == rec3p1RHSsumSplit2
```

Out[60]=
```
True
```

In[61]:=
```
(* GCD of the two recurrences *)
qREGCD[rec3p1LHS[[1, 1]], rec3p1RHSsumSplit1[[1, 1]], SUM[n]]
```

Out[61]=

$(-1 - q^{2n})\ \mathrm{SUM}[n] + \mathrm{SUM}[1+n]$

In[62]:=
```
(* The two sides of (3.1) satisfy the same first order recurrence
 and have the same initial conditions. Hence they are the same.*)
```

Proof or (3.2)

In[63]:=
```
(* Sums in the (3.2) identity*)
rec3p2LHSExp[n_] := Sum[q^(n1^2) q1Binomial[n, n1, q^2], {n1, 0, n}];
rec3p2RHSExp[n_] := Sum[(-1)^r q^(2 r^2) qBinomial[2 n, n - 2 r, q], {r, -n, n}]
```

In[65]:=
```
(* Checking that the initial values match *)
Table[Expand[rec3p2LHSExp[n] - rec3p2RHSExp[n]], {n, 0, 20}]
```

Out[65]=

{0, 0, 0, 0, 0, 0, 0, 0, 0, 0, 0, 0, 0, 0, 0, 0, 0, 0, 0, 0, 0}

In[66]:=
```
(* Recurrences of the sides of (3.2) *)
rec3p2LHS = qSR[qFR[q^(n1^2) qBinomial[n, n1, q^2], {n}, {n1}, {0}, {1}]]
```

Out[66]=

$\{(-1 - q^{1+2n})\ \mathrm{SUM}[n] + \mathrm{SUM}[1+n] == 0\}$

In[67]:=
```
(* The RHS sum of (3.2) needs to be split into
 two (r→ 2r and r→ 2r+1) before finding recurrences. *)
rec3p2RHSsumSplit1 =
 qSR[qFR[((-1)^r q^(2 r^2) qBinomial[2 n, n - 2 r, q]) /. r → 2 r /. (-1)^(2 r) → 1,
   {n}, {r}, {0}, {2}]]
```

Out[67]=

$\{-q^6 (-1 + q^{1+2n})^2 (1 + q^{1+2n})^2\ \mathrm{SUM}[n] + q^3 (1+q) (1+q^2) (1+q^{2+2n}) (1+q^{4+4n})\ \mathrm{SUM}[1+n] - q (1+q^2) (1 + q + q^2 + q^{3+2n} + 2 q^{4+2n} + q^{5+2n} + q^{6+4n} + q^{7+4n} + q^{8+4n})\ \mathrm{SUM}[2+n] + (1+q) (1+q^2) (1+q^{4+2n})\ \mathrm{SUM}[3+n] - \mathrm{SUM}[4+n] == 0\}$

In[68]:=
```
rec3p2RHSsumSplit2 =
 qSR[qFR[((-1)^r q^(2 r^2) qBinomial[2 n, n - 2 r, q]) /. r → 2 r + 1 /. (-1)^(2 r + 1) → -1,
   {n}, {r}, {0}, {2}]]
```

Out[68]=

$\{-q^6 (-1 + q^{1+2n})^2 (1 + q^{1+2n})^2\ \mathrm{SUM}[n] + q^3 (1+q) (1+q^2) (1+q^{2+2n}) (1+q^{4+4n})\ \mathrm{SUM}[1+n] - q (1+q^2) (1 + q + q^2 + q^{3+2n} + 2 q^{4+2n} + q^{5+2n} + q^{6+4n} + q^{7+4n} + q^{8+4n})\ \mathrm{SUM}[2+n] + (1+q) (1+q^2) (1+q^{4+2n})\ \mathrm{SUM}[3+n] - \mathrm{SUM}[4+n] == 0\}$

In[69]:=
```
rec3p2RHSsumSplit1 == rec3p2RHSsumSplit2
```

Out[69]=

True

In[70]:=
```
(* GCD of the two recurrences *)
qREGCD[rec3p2LHS[[1, 1]], rec3p2RHSsumSplit1[[1, 1]], SUM[n]]
```

Out[70]=

$(-1 - q^{1+2n})\ \mathrm{SUM}[n] + \mathrm{SUM}[1+n]$

In[71]:=
```
(* The two sides of (3.2) satisfy the same first order recurrence
 and have the same initial conditions. Hence they are the same.*)
```

A Substitution of the recurrences

In[72]:=
```
(* Recurrences of CP(2,0) and CP(1,1) from the recurrences of (3.1) and (3.2) *)
```

In[73]:=
```
qRESubstitute[(-1 - q^(2 n)) SUM[n] + SUM[1 + n], SUM[n], 1 / qPochhammer[q, q, 2 n]]
```

Out[73]=

$$\left(-1-q^{2n}\right) \mathrm{SUM}[n] + \left(-1+q^{1+n}\right)\left(1+q^{1+n}\right)\left(-1+q^{1+2n}\right) \mathrm{SUM}[1+n]$$

In[74]:=
```
qRESubstitute[(-1 - q^(1+2 n)) SUM[n] + SUM[1 + n], SUM[n], 1 / qPochhammer[q, q, 2 n]]
```

Out[74]=

$$\left(-1-q^{1+2n}\right) \mathrm{SUM}[n] + \left(-1+q^{1+n}\right)\left(1+q^{1+n}\right)\left(-1+q^{1+2n}\right) \mathrm{SUM}[1+n]$$

Proof of Theorem 4.3

How to find the expression using qFunctions:

In[75]:=
```
(*Recurrence and the initial conditions of P_{2,1}(n) *)
recThm4p2 = P21[n] - (1 + q^(2 n - 1) + q^(2 n - 2)) P21[n - 1] + q^(4 n - 5) P21[n - 2];
```

In[76]:=
```
P21Inits = {1, 1 + q};(*P_{2,1}(0) =1,P_{2,1}(1) =1+q*)
```

In[77]:=
```
(* Generate more initial conditions of P_{2,1}*)
qreListP21 = qREToList[recThm4p2, P21[n], {0, P21Inits}, 20];
```

In[78]:=
```
(* Find a polynomial fitting of the list using qBinomial functions *)
FitqBinomialRepresentation[qreListP21, n, 2 n, n, q]
```

Out[78]=

$$\mathrm{qBinomial}[2n, n, q] - q^{2}\,\mathrm{qBinomial}[2n, 2+n, q] - q^{3}\,\mathrm{qBinomial}[2n, 3+n, q] + \left(q^{9}+q^{11}\right)\mathrm{qBinomial}[2n, 5+n, q] - q^{21}\,\mathrm{qBinomial}[2n, 7+n, q] - q^{24}\,\mathrm{qBinomial}[2n, 8+n, q] + \left(q^{38}+q^{42}\right)\mathrm{qBinomial}[2n, 10+n, q] - q^{60}\,\mathrm{qBinomial}[2n, 12+n, q] - q^{65}\,\mathrm{qBinomial}[2n, 13+n, q] + \left(q^{87}+q^{93}\right)\mathrm{qBinomial}[2n, 15+n, q] - q^{119}\,\mathrm{qBinomial}[2n, 17+n, q] - q^{126}\,\mathrm{qBinomial}[2n, 18+n, q]$$

In[79]:=
```
(* The above polynomial fitting can be formulated as follows: *)
(* Bilateral sum expression*)P21Exp[n_] :=
 Sum[q^(10 j^2 + j) qBinomial[2 n, n + 5 j, q], {j, -Ceiling[n / 5], Ceiling[n / 5]}] - Sum[
   q^(10 j^2 + 11 j + 3) qBinomial[2 n, n + 5 j + 3, q], {j, -Ceiling[n / 5], Ceiling[n / 5]}]
```

In[80]:=
```
(* Checking that the initial conditions match *)
Table[P21Exp[i - 1] - qreListP21[[i]], {i, 20}] // Expand
```

Out[80]=
```
{0, 0, 0, 0, 0, 0, 0, 0, 0, 0, 0, 0, 0, 0, 0, 0, 0, 0, 0, 0}
```

Combining P21Exp to the (1.1) expression

In[81]:=
```
P21ExpCombined[n_] := Sum[
  (-1)^r q^(r (5 r + 1) / 2) qBinomial[2 n, n - 5 r / 2 +  ((-1)^r - 1) / 4 , q], {r, -n, n}]
```

In[82]:=
```
Table[P21ExpCombined[i - 1] - qreListP21[[i]], {i, 20}] // Expand
```

Out[82]=
```
{0, 0, 0, 0, 0, 0, 0, 0, 0, 0, 0, 0, 0, 0, 0, 0, 0, 0, 0, 0}
```

How to prove that P21 (with the expression (4.2)) satisfies the recurrence (4.1)

In[83]:=
```
(* P21Exp above has two sums. We find and
 prove recurrences satisfied by these individually. *)
rec1 = qSR[qFR[q^(10 j^2 + j) qBinomial[2 n, n - 5 j, q], {n}, {j}, {0}, {2}]]
```

Out[83]=

$$\{-q^{18+8n}(-1+q^{1+n})(1+q^{1+n})(-1+q^{1+2n})\,\mathrm{SUM}[n] + q^{13+4n}(1+q^2-q^{6+4n}-q^{7+4n}-q^{8+4n}-q^{9+4n}+q^{7+6n}+q^{8+6n}+q^{9+6n}+q^{10+6n}+q^{11+6n}+q^{12+6n})\,\mathrm{SUM}[1+n] - q^6(1+q^{3+2n}+q^{4+2n}+q^{6+4n}+q^{7+4n}+q^{8+4n}+q^{9+6n}+q^{10+6n}+q^{11+6n}+q^{12+6n}+q^{12+8n}+q^{13+8n}+2q^{14+8n}+2q^{15+8n}+3q^{16+8n}+2q^{17+8n}+2q^{18+8n}+q^{19+8n}+q^{20+8n})\,\mathrm{SUM}[2+n] + q^3(1+q)(1+q^2+q^{4+2n}+q^{5+2n}+2q^{6+2n}+q^{8+2n}+q^{8+4n}+q^{9+4n}+2q^{10+4n}+q^{11+4n}+2q^{12+4n}+q^{14+4n}+q^{12+6n}+2q^{14+6n}+q^{15+6n}+2q^{16+6n}+q^{17+6n}+2q^{18+6n}+q^{20+6n})\,\mathrm{SUM}[3+n] - q(1+q+2q^2+q^3+q^4+q^{5+2n}+2q^{6+2n}+3q^{7+2n}+3q^{8+2n}+2q^{9+2n}+2q^{10+2n}+q^{11+2n}+q^{10+4n}+q^{11+4n}+2q^{12+4n}+2q^{13+4n}+3q^{14+4n}+2q^{15+4n}+2q^{16+4n}+q^{17+4n}+q^{18+4n})\,\mathrm{SUM}[4+n] + (1+q)(1+q^2+q^{6+2n}+q^{8+2n}+q^{10+2n})\,\mathrm{SUM}[5+n] - \mathrm{SUM}[6+n] == 0\}$$

In[84]:=
```
rec2 = qSR[qFR[-q^(10 j^2 + 11 j + 3) qBinomial[2 n, n - 5 j - 3, q], {n}, {j}, {0}, {2}]]
```

Out[84]=

$$\{-q^{18+8n}(-1+q^{1+n})(1+q^{1+n})(-1+q^{1+2n})\,\mathrm{SUM}[n] + q^{13+4n}(1+q^2-q^{6+4n}-q^{7+4n}-q^{8+4n}-q^{9+4n}+q^{7+6n}+q^{8+6n}+q^{9+6n}+q^{10+6n}+q^{11+6n}+q^{12+6n})\,\mathrm{SUM}[1+n] - q^6(1+q^{3+2n}+q^{4+2n}+q^{6+4n}+q^{7+4n}+q^{8+4n}+q^{9+6n}+q^{10+6n}+q^{11+6n}+q^{12+6n}+q^{12+8n}+q^{13+8n}+2q^{14+8n}+2q^{15+8n}+3q^{16+8n}+2q^{17+8n}+2q^{18+8n}+q^{19+8n}+q^{20+8n})\,\mathrm{SUM}[2+n] + q^3(1+q)(1+q^2+q^{4+2n}+q^{5+2n}+2q^{6+2n}+q^{8+2n}+q^{8+4n}+q^{9+4n}+2q^{10+4n}+q^{11+4n}+2q^{12+4n}+q^{14+4n}+q^{12+6n}+2q^{14+6n}+q^{15+6n}+2q^{16+6n}+q^{17+6n}+2q^{18+6n}+q^{20+6n})\,\mathrm{SUM}[3+n] - q(1+q+2q^2+q^3+q^4+q^{5+2n}+2q^{6+2n}+3q^{7+2n}+3q^{8+2n}+2q^{9+2n}+2q^{10+2n}+q^{11+2n}+q^{10+4n}+q^{11+4n}+2q^{12+4n}+2q^{13+4n}+3q^{14+4n}+2q^{15+4n}+2q^{16+4n}+q^{17+4n}+q^{18+4n})\,\mathrm{SUM}[4+n] + (1+q)(1+q^2+q^{6+2n}+q^{8+2n}+q^{10+2n})\,\mathrm{SUM}[5+n] - \mathrm{SUM}[6+n] == 0\}$$

In[85]:=
```
(* These two recurrences are the same *)
rec1 == rec2
```

Out[85]=
```
True
```

In[86]:=
```
(* The proven recurrences are not necesarily the minimal recurrences. We can prove
 that the P21Exp satisfies recThm4p2 by finding the GCD of the recurrence
 recThm4p2 and the proven recurrences (rec1, rec2) (by qFunctions-qREGCD. *)
qREGCD[recThm4p2, rec1[[1, 1]] /. SUM → P21, P21[n]]
```

Out[86]=

$$q^{3+4n}\,\mathrm{P21}[n] + (-1-q^{2+2n}-q^{3+2n})\,\mathrm{P21}[1+n] + \mathrm{P21}[2+n]$$

In[87]:=
```
(* The GCD of the recurrences is the recurrence of recThm4p2,
which proves that P21Exp[n] satisfies the recurrence of
 recThm4p2 (with n→ n+2; qFunctions prefers forward shifts).
  We already showed that P21Ext[n] and the
 initial values of the recurrence recThm4p2 match.
  Hence, P21[n] has the formula P21Exp[n].*)
```

Proof of Theorem 4.6

Checking Initial Conditions of (4.5)

In[88]:=
```
(*Recurrence and the initial conditions of P_{3,0}(n) *)
recThm4p6 = P30[n] - (1 + q^(2 n - 3) + q^(2 n - 2)) P30[n - 1] + q^(4 n - 7) P30[n - 2];
```

In[89]:=
```
P30Inits = {1, 1} ;(*P_{3,0}(0) =P_{3,0}(1) =1, P_{3,0}(2) =1+q^2*)
```

In[90]:=
```
(* Generate more initial conditions of P_{3,0}*)
qreListP30 = qREToList[recThm4p6, P30[n], {0, P30Inits}, 20];
```

In[91]:=
```
(* Bilateral sum expression*)
P30Exp[n_] := Sum[ q^(10 j^2 + 3 j) qBinomial[2 n, n - 5 j , q], {j, -n, n}] -
  Sum[ q^(10 j^2 + 13 j + 4) qBinomial[2 n, n - 5 j - 4 , q], {j, -n, n}]
```

In[92]:=
```
Table[P30Exp[i - 1] - qreListP30[[i]], {i, 10}] // Expand
```

Out[92]=
```
{0, 0, 0, 0, 0, 0, 0, 0, 0, 0}
```

Combining P30Exp to the (1.2) expression

In[93]:=
```
P30ExpCombined[n_] := Sum[
  (-1)^r q^(r (5 r + 3) / 2) qBinomial[2 n, n - 5 r / 2 + 3 ((-1)^r - 1) / 4 , q], {r, -n, n}]
```

In[94]:=
```
Table[P30ExpCombined[i - 1] - qreListP30[[i]], {i, 20}] // Expand
```

Out[94]=
```
{0, 0, 0, 0, 0, 0, 0, 0, 0, 0, 0, 0, 0, 0, 0, 0, 0, 0, 0, 0}
```

How to prove that P30 (with the expression (4.5)) satisfies the recurrence (4.4)

In[95]:=

```
(* P30Exp above has two sums. We find and
 prove recurrences satisfied by these individually. *)
rec1 = qSR[qFR[q^(10 j^2 + 3 j) qBinomial[2 n, n - 5 j, q], {n}, {j}, {0}, {2}]]
```

Out[95]=

$$
\begin{aligned}
\{&-q^{18+8n}(-1+q^{1+n})(1+q^{1+n})(-1+q^{1+2n})\,\mathrm{SUM}[n]+\\
&q^{11+4n}(1+q^6-q^{8+4n}-q^{9+4n}-q^{10+4n}-q^{11+4n}+q^{9+6n}+q^{10+6n}+q^{11+6n}+q^{12+6n}+q^{13+6n}+q^{14+6n})\\
&\mathrm{SUM}[1+n]-\\
&q^6(1+q^{3+2n}+q^{4+2n}+q^{6+4n}+q^{7+4n}+q^{8+4n}+q^{9+6n}+q^{10+6n}+q^{11+6n}+q^{12+6n}+q^{12+8n}+\\
&\quad q^{13+8n}+2q^{14+8n}+2q^{15+8n}+3q^{16+8n}+2q^{17+8n}+2q^{18+8n}+q^{19+8n}+q^{20+8n})\,\mathrm{SUM}[2+n]+\\
&q^3(1+q)(1+q^2+q^{4+2n}+q^{5+2n}+2q^{6+2n}+q^{8+2n}+q^{8+4n}+q^{9+4n}+2q^{10+4n}+q^{11+4n}+2q^{12+4n}+\\
&\quad q^{14+4n}+q^{12+6n}+2q^{14+6n}+q^{15+6n}+2q^{16+6n}+q^{17+6n}+2q^{18+6n}+q^{20+6n})\,\mathrm{SUM}[3+n]-\\
&q(1+q+2q^2+q^3+q^4+q^{5+2n}+2q^{6+2n}+3q^{7+2n}+3q^{8+2n}+2q^{9+2n}+2q^{10+2n}+q^{11+2n}+q^{10+4n}+\\
&\quad q^{11+4n}+2q^{12+4n}+2q^{13+4n}+3q^{14+4n}+2q^{15+4n}+2q^{16+4n}+q^{17+4n}+q^{18+4n})\,\mathrm{SUM}[4+n]+\\
&(1+q)(1+q^2+q^{6+2n}+q^{8+2n}+q^{10+2n})\,\mathrm{SUM}[5+n]-\mathrm{SUM}[6+n]=0\}
\end{aligned}
$$

In[96]:=

```
rec2 = qSR[qFR[-q^(10 j^2 + 13 j + 4) qBinomial[2 n, n - 5 j - 4, q], {n}, {j}, {0}, {2}]]
```

Out[96]=

$$
\begin{aligned}
\{&-q^{18+8n}(-1+q^{1+n})(1+q^{1+n})(-1+q^{1+2n})\,\mathrm{SUM}[n]+\\
&q^{11+4n}(1+q^6-q^{8+4n}-q^{9+4n}-q^{10+4n}-q^{11+4n}+q^{9+6n}+q^{10+6n}+q^{11+6n}+q^{12+6n}+q^{13+6n}+q^{14+6n})\\
&\mathrm{SUM}[1+n]-\\
&q^6(1+q^{3+2n}+q^{4+2n}+q^{6+4n}+q^{7+4n}+q^{8+4n}+q^{9+6n}+q^{10+6n}+q^{11+6n}+q^{12+6n}+q^{12+8n}+\\
&\quad q^{13+8n}+2q^{14+8n}+2q^{15+8n}+3q^{16+8n}+2q^{17+8n}+2q^{18+8n}+q^{19+8n}+q^{20+8n})\,\mathrm{SUM}[2+n]+\\
&q^3(1+q)(1+q^2+q^{4+2n}+q^{5+2n}+2q^{6+2n}+q^{8+2n}+q^{8+4n}+q^{9+4n}+2q^{10+4n}+q^{11+4n}+2q^{12+4n}+\\
&\quad q^{14+4n}+q^{12+6n}+2q^{14+6n}+q^{15+6n}+2q^{16+6n}+q^{17+6n}+2q^{18+6n}+q^{20+6n})\,\mathrm{SUM}[3+n]-\\
&q(1+q+2q^2+q^3+q^4+q^{5+2n}+2q^{6+2n}+3q^{7+2n}+3q^{8+2n}+2q^{9+2n}+2q^{10+2n}+q^{11+2n}+q^{10+4n}+\\
&\quad q^{11+4n}+2q^{12+4n}+2q^{13+4n}+3q^{14+4n}+2q^{15+4n}+2q^{16+4n}+q^{17+4n}+q^{18+4n})\,\mathrm{SUM}[4+n]+\\
&(1+q)(1+q^2+q^{6+2n}+q^{8+2n}+q^{10+2n})\,\mathrm{SUM}[5+n]-\mathrm{SUM}[6+n]=0\}
\end{aligned}
$$

In[97]:=

```
(* These two recurrences are the same *)
rec1 == rec2
```

Out[97]=

True

In[98]:=

```
(* The proven recurrences are not necesarily the minimal recurrences. We can prove
 that the P30Exp satisfies recThm4p6 by finding the GCD of the recurrence
 recThm4p6 and the proven recurrences (rec1, rec2) (by qFunctions-qREGCD. *)
qREGCD[recThm4p6, rec1[[1, 1]] /. SUM → P30, P30[n]]
```

Out[98]=

$$q^{1+4n}\,\mathrm{P30}[n]+(-1-q^{1+2n}-q^{2+2n})\,\mathrm{P30}[1+n]+\mathrm{P30}[2+n]$$

In[99]:=
```
(* The GCD of the recurrences is the recurrence of recThm4p6,
which proves that P30Exp[n] satisfies the recurrence of
 recThm4p6 (with n→ n+2; qFunctions prefers forward shifts).
  We already showed that P30Exp[n] and the
 initial values of the recurrence recThm4p5 match.
  Hence, P30[n] has the formula P30Exp[n].*)
```

Manifestly Positive expression for P_{3,0}(n) (equation (4.8))

In[100]:=
```
(* Manifestly positive expression: *)
P30Pos[n_] := Sum[q^(j^2 + j) q1Binomial[2 n - j - 2, j, q], {j, 0, n}]
```

In[101]:=
```
(* Numerical checks against the P30 formula given by (4.5) *)
Table[Expand[P30Pos[n] - P30ExpCombined[n]], {n, 0, 10}]
```

Out[101]=
```
{0, 0, 0, 0, 0, 0, 0, 0, 0, 0, 0}
```

In[102]:=
```
(* Recalling the recurrence of P_{3,0}(n)*)
Collect[recThm4p5 /. {n → n + 2}, P30[_], Factor]
```

Out[102]=
```
recThm4p5
```

In[103]:=
```
(* Finding and proving the recurrence for the manifestly
 positive version of P_{3,0}(n), which is the same as the
 recurrence above (and they satisfy the same initial conditions). *)
recP30Pos = qSR[qFR[q^(j^2 + j) qBinomial[2 n - j - 2, j, q], {n}, {j}, {0}, {1}]]
```

Out[103]=

$$\left\{-q^{1+4n}\,\mathrm{SUM}[n] + \left(1 + q^{1+2n} + q^{2+2n}\right)\mathrm{SUM}[1+n] - \mathrm{SUM}[2+n] == 0\right\}$$

Proof of Theorem 5.2

Checking Initial Conditions of (5.1)

In[104]:=
```
(*Recurrence and the initial conditions of P_{4,0}(n) *)
recThm5p2 = P40[n] - (1 + q^(2 n - 4) + q^(2 n - 3) + q^(2 n - 2)) P40[n - 1] +
    (q^(4 n - 7) + q^(4 n - 8) + q^(4 n - 9)) P40[n - 2] +
    (q^(4 n - 10) - q^(6 n - 15)) P40[n - 3];
```

In[105]:=
```
P40Inits = {1, 1, 1 + q^2};(*P_{4,0}(0) =P_{4,0}(1) =1, P_{4,0}(2) =1+q^2*)
```

In[106]:=
```
(* Generate more initial conditions of P_{4,0}*)
qreListP40 = qREToList[recThm5p2, P40[n], {0, P40Inits}, 20];
```

In[107]:=
```
(* Bilateral sum expression*)P40Exp[n_] :=
 Sum[ (-1)^r q^(r (3 r + 2)) qBinomial[2 n, n - 3 r + (-1)^r - 1 , q], {r, -n, n}]
```

In[108]:=
```
Table[P40Exp[i - 1] - qreListP40[[i]], {i, 20}] // Expand
```

Out[108]=
```
{0, 0, 0, 0, 0, 0, 0, 0, 0, 0, 0, 0, 0, 0, 0, 0, 0, 0, 0, 0}
```

How to prove (5.1)

In[109]:=
```
(* We split P40Exp with r→
 2r and r→ 2r+1 and find (and prove) recurrences satisfied by these individually. *)
rec1 = qSR[qFR[((-1)^r q^(r (3 r + 2)) qBinomial[2 n, n - 3 r + (-1)^r - 1 , q]) /. r → 2 r /.
      (-1)^(2 r) → 1, {n}, {r}, {0}, {2}]];
```

In[110]:=
```
rec2 =
  qSR[qFR[((-1)^r q^(r (3 r + 2)) qBinomial[2 n, n - 3 r + (-1)^r - 1 , q]) /. r → 2 r + 1 /.
      (-1)^(2 r + 1) → -1, {n}, {r}, {0}, {2}]];
```

In[111]:=
```
(* These two recurrences are the same *)
rec1 == rec2
```

Out[111]=
```
True
```

In[112]:=
```
(* Compare these recurrences with the recurrence satisfied by P40Exp[n]
 (by qFunctions-qREGCD. *)qREGCD[recThm5p2, rec1[[1, 1]] /. SUM → P40, P40[n]]
```

Out[112]=

$$-q^{2+4n}\left(-1+q^{1+2n}\right)\text{P40}[n]+q^{3+4n}\left(1+q+q^2\right)\text{P40}[1+n]+\left(-1-q^{2+2n}-q^{3+2n}-q^{4+2n}\right)\text{P40}[2+n]+\text{P40}[3+n]$$

In[113]:=
```
(* The GCD of the recurrences is the recurrence of recThm5p2,
which proves that P40Exp[n] satisfies the recurrence of
 recThm5p2 (with n→ n+3; qFunctions prefers forward shifts).
  We already showed that P40Exp[n] and the
 initial values of the recurrence recThm5p2 match.
  Hence, equation (5.1) holds.*)
```

Manifestly Positive expression for P_{4,0}(n) - Theorem 5.3

In[114]:=
```
(* Manifestly positive expression: *)
P40Pos[n_] := Sum[q^(n1^2 + n1 + n2^2 + n2) q1Binomial[n - n1 - 2, n2, q^2] ×
   q1Binomial[2 n - n1 - n2 - 2, n1 - n2, q], {n1, 0, n}, {n2, 0, n}]
```

In[115]:=
```
(* Numerical checks against the P40 formula given by Theorem 5.3 *)
Table[Expand[P40Pos[n] - P40Exp[n]], {n, 0, 10}]
```
Out[115]=
```
{0, 0, 0, 0, 0, 0, 0, 0, 0, 0, 0}
```
In[116]:=
```
(* Recalling the recurrence of P_{4,0}(n)*)
Collect[recThm5p2 /. {n → n + 3}, P40[_], Factor]
```
Out[116]=

$$-q^{2+4n}\left(-1+q^{1+2n}\right) P40[n] + q^{3+4n}\left(1+q+q^2\right) P40[1+n] + \left(-1-q^{2+2n}-q^{3+2n}-q^{4+2n}\right) P40[2+n] + P40[3+n]$$

In[117]:=
```
(* Finding and proving the recurrence for the manifestly
 positive version of P_{4,0}(n), which is not same as the
 recurrence above (and they satisfy the same initial conditions). *)
recP40Pos = qSR[qFR[q^(n1^2 + n1 + n2^2 + n2) qBinomial[n - n1 - 2, n2, q^2] ×
    qBinomial[2 n - n1 - n2 - 2, n1 - n2, q], {n}, {n1, n2}, {0}, {1, 1}]]
```
Out[117]=

$$\Big\{-q^{2n}\left(-1+q^{1+2n}\right) SUM[n] + \left(1+q^{1+2n}+q^{2+2n}\right) SUM[1+n] - SUM[2+n] == 0,$$
$$q^{2+4n}\left(-1+q^{1+2n}\right) SUM[n] - q^{3+4n}\left(1+q+q^2\right) SUM[1+n] + \left(1+q^{2+2n}+q^{3+2n}+q^{4+2n}\right) SUM[2+n] - SUM[3+n] == 0,$$
$$-q^{3+4n}(1+q)\left(-1+q^{1+2n}\right) SUM[n] + q^{2+2n}\left(1+q^{2+2n}+q^{3+2n}+2q^{4+2n}+q^{5+2n}\right) SUM[1+n] + \left(1-q-q^2-q^{4+2n}-2q^{5+2n}-q^{6+2n}\right) SUM[2+n] + \left(-1+q+q^2\right) SUM[3+n] == 0\Big\}$$

In[118]:=
```
(* Notice that qMultiSums finds (and proves) three recurrences here. The
 middle one mathces the original recurrence of P_{4,0}(n). We already
 checked that their initial values match. Hence,Thm 5.3 is proven. *)
```

Proof of Theorem 5.5

Checking Initial Conditions of (5.3)

In[119]:=
```
(*Recurrence and the initial conditions of P_{3,1}(n) *)
recThm5p5 = P31[n] - (1 + q^(2 n - 3) + q^(2 n - 2) + q^(2 n - 1)) P31[n - 1] +
   (q^(4 n - 5) + q^(4 n - 6) + q^(4 n - 7)) P31[n - 2] +
   (q^(4 n - 8) - q^(6 n - 12)) P31[n - 3];
```
In[120]:=
```
P31Inits = {1, 1 + q, 1 + q + q^2 + q^3 + q^4};(*P_{3,1}(0) =1,
P_{3,1}(1) =1+q, P_{3,1}(2) =1+q+q^2+q^4+q^4*)
```
In[121]:=
```
(* Generate more initial conditions of P_{3,1}*)
qreListP31 = qREToList[recThm5p5, P31[n], {0, P31Inits}, 20];
```
In[122]:=
```
(* Bilateral sum expression*)P31Exp[n_] :=
 Sum[ (-1)^r q^(r (3 r + 1)) qBinomial[2 n, n - 3 r + ((-1)^r - 1) / 2 , q], {r, -n, n}]
```

In[123]:=
```
Table[P31Exp[i - 1] - qreListP31⟦i⟧, {i, 20}] // Expand
```
Out[123]=
```
{0, 0, 0, 0, 0, 0, 0, 0, 0, 0, 0, 0, 0, 0, 0, 0, 0, 0, 0, 0}
```

How to prove (5.3)

In[124]:=
```
(* We split P31Exp with r→
 2r and r→ 2r+1 and find (and prove) recurrences satisfied by these individually. *)
rec1 = qSR[qFR[((-1)^r q^(r (3 r + 1)) qBinomial[2 n, n - 3 r + ((-1)^r - 1) / 2, q]) /.
      r → 2 r /. (-1)^(2 r) → 1, {n}, {r}, {0}, {2}]];
```
In[125]:=
```
rec2 = qSR[
   qFR[((-1)^r q^(r (3 r + 1)) qBinomial[2 n, n - 3 r + ((-1)^r - 1) / 2, q]) /. r → 2 r + 1 /.
     (-1)^(2 r + 1) → -1, {n}, {r}, {0}, {2}]];
```
In[126]:=
```
(* These two recurrences are the same *)
rec1 == rec2
```
Out[126]=
```
True
```
In[127]:=
```
(* Compare these recurrences with the recurrence
 satisfied by P31Exp[n] (by qFunctions-qREGCD. *)
qREGCD[recThm5p5, rec1⟦1, 1⟧ /. SUM → P31, P31[n]]
```
Out[127]=

$$-q^{4+4n}\left(-1+q^{1+n}\right)\left(1+q^{1+n}\right)\text{P31}[n]+q^{5+4n}\left(1+q+q^2\right)\text{P31}[1+n]+\left(-1-q^{3+2n}-q^{4+2n}-q^{5+2n}\right)\text{P31}[2+n]+\text{P31}[3+n]$$

In[128]:=
```
(* The GCD of the recurrences is the recurrence of recThm5p5,
which proves that P31Exp[n] satisfies the recurrence of
 recThm5p5 (with n→ n+3; qFunctions prefers forward shifts).
  We already showed that P31Exp[n] and the
 initial values of the recurrence recThm5p2 match.
  Hence, equation (5.3) holds.*)
```

Manifestly Positive expression for P_{3,1}(n) - Theorem 5.6

In[129]:=
```
(* Manifestly positive expression: *)
P31Pos[n_] := Sum[q^(n1^2 + n2^2 + n2) q1Binomial[n - n1 - 1, n2, q^2] ×
   q1Binomial[2 n - n1 - n2, n1 - n2, q], {n1, 0, n}, {n2, 0, n}]
```
In[130]:=
```
(* Numerical checks against the P31 formula given by Theorem 5.3 *)
Table[Expand[P31Pos[n] - P31Exp[n]], {n, 0, 10}]
```
Out[130]=
```
{0, 0, 0, 0, 0, 0, 0, 0, 0, 0, 0}
```

In[131]:=
```
(* Recalling the recurrence of P_{3,1}(n)*)
Collect[recThm5p5 /. {n → n + 3}, P31[_], Factor]
```
Out[131]=

$$-q^{4+4n}\left(-1+q^{1+n}\right)\left(1+q^{1+n}\right)\mathrm{P31}[n]+q^{5+4n}\left(1+q+q^2\right)\mathrm{P31}[1+n]+\left(-1-q^{3+2n}-q^{4+2n}-q^{5+2n}\right)\mathrm{P31}[2+n]+\mathrm{P31}[3+n]$$

In[132]:=
```
(* Finding and proving the recurrence for the manifestly
 positive version of P_{3,1}(n), which is not same as the
 recurrence above (and they satisfy the same initial conditions). *)
recP31Pos = qSR[qFR[q^(n1^2 + n2^2 + n2) qBinomial[n - n1 - 1, n2, q^2] ×
    qBinomial[2 n - n1 - n2, n1 - n2, q], {n}, {n1, n2}, {0}, {1, 1}]]
```
Out[132]=

$$\left\{-q^{1+2n}\left(-1+q^{1+n}\right)\left(1+q^{1+n}\right)\mathrm{SUM}[n]+\left(1+q^{2+2n}+q^{3+2n}\right)\mathrm{SUM}[1+n]-\mathrm{SUM}[2+n]==0,\right.$$
$$q^{4+4n}\left(-1+q^{1+n}\right)\left(1+q^{1+n}\right)\mathrm{SUM}[n]-q^{5+4n}\left(1+q+q^2\right)\mathrm{SUM}[1+n]+\left(1+q^{3+2n}+q^{4+2n}+q^{5+2n}\right)\mathrm{SUM}[2+n]-\mathrm{SUM}[3+n]==0,$$
$$-q^{5+4n}(1+q)\left(-1+q^{1+n}\right)\left(1+q^{1+n}\right)\mathrm{SUM}[n]+q^{3+2n}\left(1+q^{3+2n}+q^{4+2n}+2q^{5+2n}+q^{6+2n}\right)\mathrm{SUM}[1+n]+$$
$$\left.\left(1-q-q^2-q^{5+2n}-2q^{6+2n}-q^{7+2n}\right)\mathrm{SUM}[2+n]+\left(-1+q+q^2\right)\mathrm{SUM}[3+n]==0\right\}$$

In[133]:=
```
(* Notice that qMultiSums finds (and proves) three recurrences here. The
 middle one mathces the original recurrence of P_{3,1}(n). We already
 checked that their initial values match. Hence,Thm 5.6 is proven. *)
```

Proof of Theorem 5.8

Checking Initial Conditions of (5.4)

In[134]:=
```
(*Recurrence and the initial conditions of P_{2,2}(n) *)
recThm5p8 =
  P22[n] - (1 + q^(2 n - 2) + q^(2 n - 1)) P22[n - 1] - (q^(2 n - 2) - q^(4 n - 5)) P22[n - 2];
```
In[135]:=
```
P22Inits = {1, 1 + q} ;(*P_{2,2}(0) =1, P_{2,2}(1) =1+q*)
```
In[136]:=
```
(* Generate more initial conditions of P_{3,1}*)
qreListP22 = qREToList[recThm5p8, P22[n], {0, P22Inits}, 20];
```
In[137]:=
```
(* Bilateral sum expression*)
P22Exp[n_] := Sum[ (-1)^r q^(3 r^2) qBinomial[2 n, n - 3 r, q], {r, -n, n}]
```
In[138]:=
```
Table[P22Exp[i - 1] - qreListP22[[i]], {i, 20}] // Expand
```
Out[138]=
```
{0, 0, 0, 0, 0, 0, 0, 0, 0, 0, 0, 0, 0, 0, 0, 0, 0, 0, 0, 0}
```

How to prove (5.4)

In[139]:=

```
(* We split P22Exp with r→
 2r and r→ 2r+1 and find (and prove) recurrences satisfied by these individually. *)
rec1 = qSR[qFR[((-1)^r q^(3 r^2) qBinomial[2 n, n - 3 r, q]) /. r → 2 r /. (-1)^(2 r) → 1,
    {n}, {r}, {0}, {2}]];
```

In[140]:=

```
rec2 = qSR[qFR[((-1)^r q^(3 r^2) qBinomial[2 n, n - 3 r, q]) /. r → 2 r + 1 /.
     (-1)^(2 r + 1) → -1, {n}, {r}, {0}, {2}]];
```

In[141]:=

```
(* These two recurrences are the same *)
rec1 == rec2
```

Out[141]=

True

In[142]:=

```
(* Compare these recurrences with the recurrence
 satisfied by P22Exp[n] (by qFunctions-qREGCD. *)
qREGCD[recThm5p8, rec1[[1, 1]] /. SUM → P22, P22[n]]
```

Out[142]=

$$q^{2+2n}\left(-1+q^{1+2n}\right) P22[n] + \left(-1-q^{2+2n}-q^{3+2n}\right) P22[1+n] + P22[2+n]$$

In[143]:=

```
(* The GCD of the recurrences is the recurrence of recThm5p8,
which proves that P22Exp[n] satisfies the recurrence of
 recThm5p8 (with n→ n+2; qFunctions prefers forward shifts).
  We already showed that P22Exp[n] and the
 initial values of the recurrence above recThm5p8 match.
  Hence, equation (5.4) holds.*)
```

Manifestly Positive expression for P_{2,2}(n) - Theorem 5.9

In[144]:=

```
(* Manifestly positive expression: *)
P22Pos[n_] := Sum[q^(n1^2 + n2^2) q1Binomial[n - n1, n2, q^2] ×
   q1Binomial[2 n - n1 - n2, n1 - n2, q], {n1, 0, n}, {n2, 0, n}]
```

In[145]:=

```
(* Numerical checks against the P40 formula given by Theorem 5.3 *)
Table[Expand[P22Pos[n] - P22Exp[n]], {n, 0, 10}]
```

Out[145]=

{0, 0, 0, 0, 0, 0, 0, 0, 0, 0, 0}

In[146]:=

```
(* Recalling the recurrence of P_{2,2}(n)*)
Collect[recThm5p8 /. {n → n + 2}, P22[_], Factor]
```

Out[146]=

$$q^{2+2n}\left(-1+q^{1+2n}\right) P22[n] + \left(-1-q^{2+2n}-q^{3+2n}\right) P22[1+n] + P22[2+n]$$

In[147]:=

```
(* Finding and proving the recurrence for the manifestly
 positive version of P_{2,2}(n), which is not same as the
 recurrence above (and they satisfy the same initial conditions). *)
```

In[148]:=

```
recP31Pos =
 qSR[qFR[q^(n1^2 + n2^2) qBinomial[n - n1, n2, q^2] × qBinomial[2 n - n1 - n2, n1 - n2, q],
   {n}, {n1, n2}, {0}, {1, 0}]]
```

Out[148]=

$\{-q^{2+2n} (-1 + q^{1+2n}) \text{SUM}[n] + (1 + q^{2+2n} + q^{3+2n}) \text{SUM}[1 + n] - \text{SUM}[2 + n] == 0\}$

In[149]:=

```
(* Notice that qMultiSums finds (and proves) the
 original recurrence of P_{2,2}(n). We already checked that
 their initial values match. Hence,Thm 5.9 is proven. *)
```

Proof of Theorem 6.2

Checking Initial Conditions of (6.1)

In[150]:=

```
(*Recurrence and the initial conditions of P_{5,0}(n) *)
recThm6p2 = P50[n] - (1 + q^(2 n - 5) + q^(2 n - 4) + q^(2 n - 3) + q^(2 n - 2)) P50[n - 1] +
   q^(4 n - 11) (1 + q + 2 q^2 + q^3 + q^4) P50[n - 2] +
   (q^(4 n - 12) (1 + q + q^2) - q^(6 n - 18) (1 + q + q^2 + q^3)) P50[n - 3] +
   q^(4 n - 13) (1 - q^(2 n - 6)) (1 - q^(2 n - 7)) P50[n - 4];
```

In[151]:=

```
P50Inits = {1, 1, 1 + q^2, 1 + q^2 + q^3 + q^4 + q^6} ;
(*P_{5,0}(0) =1, P_{5,0}(1) =1, ...*)
```

In[152]:=

```
(* Generate more initial conditions of P_{5,0}*)
qreListP50 = qREToList[recThm6p2, P50[n], {0, P50Inits}, 20];
```

In[153]:=

```
(* Bilateral sum expression*)P50Exp[n_] := Sum[
  (-1)^r q^(r (7 r + 5) / 2) qBinomial[2 n, n - 7 r / 2 + 5 ((-1)^r - 1) / 4, q], {r, -n, n}]
```

In[154]:=

```
Table[P50Exp[i - 1] - qreListP50[[i]], {i, 20}] // Expand
```

Out[154]=

```
{0, 0, 0, 0, 0, 0, 0, 0, 0, 0, 0, 0, 0, 0, 0, 0, 0, 0, 0, 0}
```

How to prove (6.1)

In[155]:=

```
(* We split P50Exp with r→ 2r and r→
  2r+1 and find (and prove) recurrences satisfied by these individually. *)rec1 =
  qSR[qFR[ (-1)^r q^(r (7 r + 5) / 2) qBinomial[2 n, n - 7 r / 2 + 5 ((-1)^r - 1) / 4, q] /.
      r → 2 r /. (-1)^(2 r) → 1, {n}, {r}, {0}, {2}]];
```

In[156]:=

```
rec2 =
  qSR[qFR[ (-1) ^r q^ (r (7 r + 5) / 2) qBinomial[2 n, n - 7 r / 2 + 5 ((-1) ^r - 1) / 4, q] /.
      r → 2 r + 1 /. (-1) ^ (2 r + 1) → -1, {n}, {r}, {0}, {2}]];
```

In[157]:=

```
(* These two recurrences are the same *)
rec1 == rec2
```

Out[157]=

```
True
```

In[158]:=

```
(* Compare these recurrences with the recurrence
 satisfied by P50Exp[n] (by qFunctions-qREGCD. *)
qREGCD[recThm6p2, rec1[[1, 1]] /. SUM → P50, P50[n]]
```

Out[158]=

$$q^{3+4n}\left(-1+q^{1+n}\right)\left(1+q^{1+n}\right)\left(-1+q^{1+2n}\right)\text{P50}[n] - q^{4+4n}\left(-1-q-q^2+q^{2+2n}+q^{3+2n}+q^{4+2n}+q^{5+2n}\right)\text{P50}[1+n] + q^{5+4n}\left(1+q^2\right)\left(1+q+q^2\right)\text{P50}[2+n] + \left(-1-q^{3+2n}-q^{4+2n}-q^{5+2n}-q^{6+2n}\right)\text{P50}[3+n] + \text{P50}[4+n]$$

In[159]:=

```
(* The GCD of the recurrences is the recurrence of recThm6p2,
which proves that P50Exp[n] satisfies the recurrence of
 recThm6p2 (with n→ n+4; qFunctions prefers forward shifts).
  We already showed that P50Exp[n] and the
 initial values of the recurrence recThm5p2 match.
  Hence,equation (6.1) holds.*)
```

Proof of Theorem 6.4

Checking Initial Conditions of (6.2)

In[160]:=

```
(*Recurrence and the initial conditions of P_{4,1}(n) *)
recThm6p4 = P41[n] - (1 + q^ (2 n - 4) + q^ (2 n - 3) + q^ (2 n - 2) + q^ (2 n - 1)) P41[n - 1] +
   q^ (4 n - 9) (1 + q^2) (1 + q + q^2) P41[n - 2] +
   (q^ (4 n - 10) (1 + q + q^2) - q^ (6 n - 15) (1 + q + q^2 + q^3)) P41[n - 3] +
   q^ (4 n - 11) (1 - q^ (2 n - 5)) (1 - q^ (2 n - 6)) P41[n - 4];
```

In[161]:=

```
P41Inits = {1, 1 + q, 1 + q + q^2 + q^3 + q^4,
   1 + q + q^2 + 2 q^3 + 2 q^4 + 2 q^5 + 2 q^6 + q^7 + q^8 + q^9} ;
 (*P_{4,1}(0) =1, P_{4,1}(1) =1+q, ...*)
```

In[162]:=

```
(* Generate more initial conditions of P_{3,1}*)
qreListP41 = qREToList[recThm6p4, P41[n], {0, P41Inits}, 20];
```

In[163]:=

```
(* Bilateral sum expression*)P41Exp[n_] := Sum[
  (-1) ^r q^ (r (7 r + 3) / 2) qBinomial[2 n, n - 7 r / 2 + 3 ((-1) ^r - 1) / 4, q], {r, -n, n}]
```

In[164]:=
```
Table[P41Exp[i - 1] - qreListP41[[i]], {i, 20}] // Expand
```
Out[164]=
```
{0, 0, 0, 0, 0, 0, 0, 0, 0, 0, 0, 0, 0, 0, 0, 0, 0, 0, 0, 0}
```

How to prove (6.2)

In[165]:=
```
(* We split P41Exp with r→ 2r and r→
  2r+1 and find (and prove) recurrences satisfied by these individually. *)rec1 =
  qSR[qFR[ (-1) ^r q^ (r (7 r + 3) / 2) qBinomial[2 n, n - 7 r / 2 + 3 ((-1) ^r - 1) / 4, q] /.
      r → 2 r /. (-1) ^ (2 r) → 1, {n}, {r}, {0}, {2}]];
```
In[166]:=
```
rec2 =
  qSR[qFR[ (-1) ^r q^ (r (7 r + 3) / 2) qBinomial[2 n, n - 7 r / 2 + 3 ((-1) ^r - 1) / 4, q] /.
      r → 2 r + 1 /. (-1) ^ (2 r + 1) → -1, {n}, {r}, {0}, {2}]];
```
In[167]:=
```
(* These two recurrences are the same *)
rec1 == rec2
```
Out[167]=
```
True
```
In[168]:=
```
(* Compare these recurrences with the recurrence
 satisfied by P41Exp[n] (by qFunctions-qREGCD. *)
qREGCD[recThm6p4, rec1[[1, 1]] /. SUM → P41, P41[n]]
```
Out[168]=

$$q^{5+4n}\left(-1+q^{1+n}\right)\left(1+q^{1+n}\right)\left(-1+q^{3+2n}\right)\mathrm{P41}[n] - q^{6+4n}\left(-1-q-q^2+q^{3+2n}+q^{4+2n}+q^{5+2n}+q^{6+2n}\right)\mathrm{P41}[1+n] + q^{7+4n}\left(1+q^2\right)\left(1+q+q^2\right)\mathrm{P41}[2+n] + \left(-1-q^{4+2n}-q^{5+2n}-q^{6+2n}-q^{7+2n}\right)\mathrm{P41}[3+n] + \mathrm{P41}[4+n]$$

In[169]:=
```
(* The GCD of the recurrences is the recurrence of recThm6p4,
which proves that P41Exp[n] satisfies the recurrence of
 recThm6p4 (with n→ n+4; qFunctions prefers forward shifts).
  We already showed that P41Exp[n] and the
 initial values of the recurrence recThm5p4 match.
  Hence,equation (6.2) holds.*)
```

Proof of Theorem 6.6

Checking Initial Conditions of (6.3)

In[170]:=
```
(*Recurrence and the initial conditions of P_{3,2}(n) *)
recThm6p6 =
  P32[n] - (1 + q^ (2 n - 4) + q^ (2 n - 3) + q^ (2 n - 2) + q^ (2 n - 1)) P32[n - 1] + q^ (2 n - 4)
     (1 - q^2 + q^ (2 n - 5) + q^ (2 n - 4) + 2 q^ (2 n - 3) + q^ (2 n - 2) + q^ (2 n - 1)) P32[n - 2] +
   q^ (4 n - 8) (2 + q - q^ (2 n - 7) - q^ (2 n - 6) - q^ (2 n - 5) - q^ (2 n - 4)) P32[n - 3] +
   q^ (4 n - 9) (1 - q^ (2 n - 6)) (1 - q^ (2 n - 7)) P32[n - 4];
```

In[171]:=

```
P32Inits = {1, 1 + q, 1 + q + 2 q^2 + q^3 + q^4,
   1 + q + 2 q^2 + 2 q^3 + 3 q^4 + 3 q^5 + 3 q^6 + 2 q^7 + q^8 + q^9} ;
 (*P_{3,2}(0) =1, P_{3,2}(1) =1+q, ...*)
```

In[172]:=

```
(* Generate more initial conditions of P_{3,2}*)
qreListP32 = qREToList[recThm6p6, P32[n], {0, P32Inits}, 20];
```

In[173]:=

```
(* Bilateral sum expression*)P32Exp[n_] :=
 Sum[ (-1)^r q^(r (7 r + 1) / 2) qBinomial[2 n, n - 7 r / 2 + ((-1)^r - 1) / 4, q], {r, -n, n}]
```

In[174]:=

```
Table[P32Exp[i - 1] - qreListP32[[i]], {i, 20}] // Expand
```

Out[174]=

```
{0, 0, 0, 0, 0, 0, 0, 0, 0, 0, 0, 0, 0, 0, 0, 0, 0, 0, 0, 0}
```

How to prove (6.3)

In[175]:=

```
(* We split P32Exp with r→
 2r and r→ 2r+1 and find (and prove) recurrences satisfied by these individually. *)
rec1 = qSR[qFR[ (-1)^r q^(r (7 r + 1) / 2) qBinomial[2 n, n - 7 r / 2 + ((-1)^r - 1) / 4, q] /.
      r → 2 r /. (-1)^(2 r) → 1, {n}, {r}, {0}, {2}]];
```

In[176]:=

```
rec2 = qSR[qFR[ (-1)^r q^(r (7 r + 1) / 2) qBinomial[2 n, n - 7 r / 2 + ((-1)^r - 1) / 4, q] /.
      r → 2 r + 1 /. (-1)^(2 r + 1) → -1, {n}, {r}, {0}, {2}]];
```

In[177]:=

```
(* These two recurrences are the same *)
rec1 == rec2
```

Out[177]=

```
True
```

In[178]:=

```
(* Compare these recurrences with the recurrence
 satisfied by P32Exp[n] (by qFunctions-qREGCD. *)
qREGCD[recThm6p6, rec1[[1, 1]] /. SUM → P32, P32[n]]
```

Out[178]=

$$q^{7+4n}\left(-1+q^{1+n}\right)\left(1+q^{1+n}\right)\left(-1+q^{1+2n}\right)\mathrm{P32}[n]-q^{8+4n}\left(-2-q+q^{1+2n}+q^{2+2n}+q^{3+2n}+q^{4+2n}\right)\mathrm{P32}[1+n]+q^{4+2n}\left(1-q^{2}+q^{3+2n}+q^{4+2n}+2\,q^{5+2n}+q^{6+2n}+q^{7+2n}\right)\mathrm{P32}[2+n]+\left(-1-q^{4+2n}-q^{5+2n}-q^{6+2n}-q^{7+2n}\right)\mathrm{P32}[3+n]+\mathrm{P32}[4+n]$$

In[179]:=

```
(* The GCD of the recurrences is the recurrence of recThm6p6,
which proves that P32Exp[n] satisfies the recurrence of
 recThm6p6 (with n→ n+4; qFunctions prefers forward shifts).
  We already showed that P32Exp[n] and the
 initial values of the recurrence recThm6p6 match.
  Hence,equation (6.3) holds.*)
```

Proofs of Theorem 1.4 with k=3 and 4

Eqn (1.5) with k=3 and i=1

In[180]:=
```
(*Recurrence of the left hand side *)
```

In[181]:=
```
summandLHS = q^(n[1]+n[1]^2+n[2]+n[2]^2) qBinomial[-2 + 2 n - n[1] - n[2], n[1] - n[2], q] ×
    qBinomial[-4 + 2 n + n[2] - 2 (n[1] + n[2]), n[2], q] /. {n[1] → n1, n[2] → n2};
```

In[182]:=
```
AbsoluteTiming[recLHS = qSR[qFR[summandLHS, {n}, {n1, n2}, {1}, {1, 0}]]]
```

Out[182]=

$$\{0.564093, \{q^{3+4n}(-1+q^{1+n})(1+q^{1+n})(-1+q^{1+2n})\,\mathrm{SUM}[n] - q^{4+4n}(-1-q-q^2+q^{2+2n}+q^{3+2n}+q^{4+2n}+q^{5+2n})\,\mathrm{SUM}[1+n] + q^{5+4n}(1+q^2)(1+q+q^2)\,\mathrm{SUM}[2+n] + (-1-q^{3+2n}-q^{4+2n}-q^{5+2n}-q^{6+2n})\,\mathrm{SUM}[3+n] + \mathrm{SUM}[4+n] == 0\}\}$$

In[183]:=
```
(* Recurrence of the right hand side *)
```

In[184]:=

$$\mathrm{summandRHS} = (-1)^r q^{\frac{1}{2} r (5+7r)} \mathrm{qBinomial}\left[2n, \frac{5}{4}\left(-1+(-1)^r\right)+n-\frac{7r}{2}, q\right];$$

In[185]:=
```
AbsoluteTiming[
 recRHSeven = qSR[qFR[summandRHS /. r → 2 r /. (-1) ^ (2 r) → 1, {n}, {r}, {2}, {2}]];]
```

Out[185]=
```
{8.44096, Null}
```

In[186]:=
```
AbsoluteTiming[recRHSodd =
   qSR[qFR[summandRHS /. r → 2 r + 1 /. (-1) ^ (2 r + 1) → -1, {n}, {r}, {2}, {2}]];]
```

Out[186]=
```
{8.01718, Null}
```

In[187]:=
```
recRHSeven == recRHSodd
```

Out[187]=
```
True
```

In[188]:=
```
(* Find the recurrence satisfied by both sides*)
```

In[189]:=
```
qREGCD[recRHSeven[[1, 1]], recLHS[[1, 1]], SUM[n]]
```

Out[189]=

$$q^{3+4n}(-1+q^{1+n})(1+q^{1+n})(-1+q^{1+2n})\,\mathrm{SUM}[n] - q^{4+4n}(-1-q-q^2+q^{2+2n}+q^{3+2n}+q^{4+2n}+q^{5+2n})\,\mathrm{SUM}[1+n] + q^{5+4n}(1+q^2)(1+q+q^2)\,\mathrm{SUM}[2+n] + (-1-q^{3+2n}-q^{4+2n}-q^{5+2n}-q^{6+2n})\,\mathrm{SUM}[3+n] + \mathrm{SUM}[4+n]$$

In[190]:=
```
(* Initial conditions are checked under
 "Multisums and Bailey's Theorem Applications" section above. *)
```

Eqn (1.5) with k=3 and i=2

In[191]:=

```
(*Recurrence of the left hand side *)
```

In[192]:=

```
summandLHS = q^(n[1]^2+n[2]+n[2]^2) qBinomial[2 n - n[1] - n[2], n[1] - n[2], q] ×
    qBinomial[-2 + 2 n + n[2] - 2 (n[1] + n[2]), n[2], q] /. {n[1] → n1, n[2] → n2};
```

In[193]:=

```
AbsoluteTiming[recLHS = qSR[qFR[summandLHS, {n}, {n1, n2}, {1}, {1, 0}]]]
```

Out[193]=

$$\{0.524374, \{q^{5+4n}(-1+q^{1+n})(1+q^{1+n})(-1+q^{3+2n})\,\mathrm{SUM}[n] - q^{6+4n}(-1-q-q^2+q^{3+2n}+q^{4+2n}+q^{5+2n}+q^{6+2n})\,\mathrm{SUM}[1+n] + q^{7+4n}(1+q^2)(1+q+q^2)\,\mathrm{SUM}[2+n] + (-1-q^{4+2n}-q^{5+2n}-q^{6+2n}-q^{7+2n})\,\mathrm{SUM}[3+n] + \mathrm{SUM}[4+n] == 0\}\}$$

In[194]:=

```
(* Recurrence of the right hand side *)
```

In[195]:=

$$\mathrm{summandRHS} = (-1)^r q^{\frac{1}{2} r (3+7r)}\,\mathrm{qBinomial}\left[2n, \frac{3}{4}\left(-1+(-1)^r\right)+n-\frac{7r}{2}, q\right];$$

In[196]:=

```
AbsoluteTiming[
 recRHSeven = qSR[qFR[summandRHS /. r → 2 r /. (-1)^(2 r) → 1, {n}, {r}, {2}, {2}]];]
```

Out[196]=

```
{8.60462, Null}
```

In[197]:=

```
AbsoluteTiming[recRHSodd =
   qSR[qFR[summandRHS /. r → 2 r + 1 /. (-1)^(2 r + 1) → -1, {n}, {r}, {2}, {2}]];]
```

Out[197]=

```
{8.24538, Null}
```

In[198]:=

```
recRHSeven == recRHSodd
```

Out[198]=

```
True
```

In[199]:=

```
(* Find the recurrence satisfied by both sides*)
```

In[200]:=

```
qREGCD[recRHSeven[[1, 1]], recLHS[[1, 1]], SUM[n]]
```

Out[200]=

$$q^{5+4n}(-1+q^{1+n})(1+q^{1+n})(-1+q^{3+2n})\,\mathrm{SUM}[n] - q^{6+4n}(-1-q-q^2+q^{3+2n}+q^{4+2n}+q^{5+2n}+q^{6+2n})\,\mathrm{SUM}[1+n] + q^{7+4n}(1+q^2)(1+q+q^2)\,\mathrm{SUM}[2+n] + (-1-q^{4+2n}-q^{5+2n}-q^{6+2n}-q^{7+2n})\,\mathrm{SUM}[3+n] + \mathrm{SUM}[4+n]$$

In[201]:=

```
(* Initial conditions are checked under
 "Multisums and Bailey's Theorem Applications" section above. *)
```

Eqn (1.5) with k=3 and i=2

In[202]:=

```
(*Recurrence of the left hand side *)
```

In[203]:=

```
summandLHS = q^(n[1]^2+n[2]^2) qBinomial[2 n - n[1] - n[2], n[1] - n[2], q] ×
    qBinomial[2 n + n[2] - 2 (n[1] + n[2]), n[2], q] /. {n[1] → n1, n[2] → n2};
```

In[204]:=

```
AbsoluteTiming[recLHS = qSR[qFR[summandLHS, {n}, {n1, n2}, {1}, {1, 0}]]]
```

Out[204]=

$$\{0.447153, \{-q^{7+4n}(-1+q^{1+n})(1+q^{1+n})(-1+q^{1+2n})\,\mathrm{SUM}[n] + q^{8+4n}(-2-q+q^{1+2n}+q^{2+2n}+q^{3+2n}+q^{4+2n})\,\mathrm{SUM}[1+n] - q^{4+2n}(1-q^2+q^{3+2n}+q^{4+2n}+2q^{5+2n}+q^{6+2n}+q^{7+2n})\,\mathrm{SUM}[2+n] + (1+q^{4+2n}+q^{5+2n}+q^{6+2n}+q^{7+2n})\,\mathrm{SUM}[3+n] - \mathrm{SUM}[4+n] == 0\}\}$$

In[205]:=

```
(* Recurrence of the right hand side *)
```

In[206]:=

$$\mathrm{summandRHS} = (-1)^r q^{\frac{1}{2} r (1+7r)}\,\mathrm{qBinomial}\left[2n, \frac{1}{4}\left(-1+(-1)^r\right)+n-\frac{7r}{2}, q\right];$$

In[207]:=

```
AbsoluteTiming[
 recRHSeven = qSR[qFR[summandRHS /. r → 2 r /. (-1) ^ (2 r) → 1, {n}, {r}, {2}, {2}]];]
```

Out[207]=

```
{9.13507, Null}
```

In[208]:=

```
AbsoluteTiming[recRHSodd =
   qSR[qFR[summandRHS /. r → 2 r + 1 /. (-1) ^ (2 r + 1) → -1, {n}, {r}, {2}, {2}]];]
```

Out[208]=

```
{8.4479, Null}
```

In[209]:=

```
recRHSeven == recRHSodd
```

Out[209]=

```
True
```

In[210]:=

```
(* Find the recurrence satisfied by both sides*)
```

In[211]:=

```
qREGCD[recRHSeven[[1, 1]], recLHS[[1, 1]], SUM[n]]
```

Out[211]=

$$q^{7+4n}(-1+q^{1+n})(1+q^{1+n})(-1+q^{1+2n})\,\mathrm{SUM}[n] - q^{8+4n}(-2-q+q^{1+2n}+q^{2+2n}+q^{3+2n}+q^{4+2n})\,\mathrm{SUM}[1+n] + q^{4+2n}(1-q^2+q^{3+2n}+q^{4+2n}+2q^{5+2n}+q^{6+2n}+q^{7+2n})\,\mathrm{SUM}[2+n] + (-1-q^{4+2n}-q^{5+2n}-q^{6+2n}-q^{7+2n})\,\mathrm{SUM}[3+n] + \mathrm{SUM}[4+n]$$

In[212]:=

```
(* Initial conditions are checked under
 "Multisums and Bailey's Theorem Applications" section above. *)
```

Eqn (1.5) with k=4 and i=1

In[213]:=

```
(*Recurrence of the left hand side *)
summandLHS = q^(n[1]+n[1]^2+n[2]+n[2]^2+n[3]+n[3]^2) qBinomial[-2 + 2 n - n[1] - n[2], n[1] - n[2], q] ×
    qBinomial[-4 + 2 n - 2 n[1] - n[2] - n[3], n[2] - n[3], q] × qBinomial[
     -6 + 2 n + n[3] - 2 (n[1] + n[2] + n[3]), n[3], q] /. {n[1] → n1, n[2] → n2, n[3] → n3};
```

In[214]:=

```
(* Running

 AbsoluteTiming[recLHS= qSR[qFR[summandLHS,{n},{n1,n2,n3},{2},{2,1,0}]]]
 takes about 8 hours on a mid level personal laptop.

  We instead use the precalculated structure set (see Riese's qMultiSum) here.

 *)
AbsoluteTiming[
 recLHS = qSR[qFR[summandLHS, {n}, {n1, n2, n3}, {0}, {0, 0, 0}, StructSet →
      {{8, 8, 0, 0}, {8, 7, 1, 0}, {8, 6, 2, 0}, {8, 5, 3, 0}, {8, 4, 4, 0}, {7, 7, 0, 0},
       {7, 6, 1, 0}, {7, 6, 0, 0}, {7, 5, 2, 0}, {7, 5, 1, 1}, {7, 5, 1, 0}, {7, 4, 3, 0},
       {7, 4, 2, 1}, {7, 4, 2, 0}, {7, 3, 3, 1}, {6, 6, 0, 0}, {6, 5, 1, 0}, {6, 5, 0, 0},
       {6, 4, 2, 0}, {6, 4, 1, 1}, {6, 4, 1, 0}, {6, 3, 3, 0}, {6, 3, 2, 1}, {6, 3, 2, 0},
       {6, 2, 2, 2}, {5, 5, 0, 0}, {5, 4, 1, 0}, {5, 4, 0, 0}, {5, 3, 2, 0}, {5, 3, 1, 1},
       {5, 3, 1, 0}, {5, 2, 2, 1}, {5, 2, 2, 0}, {4, 4, 0, 0}, {4, 3, 1, 0},
       {4, 3, 0, 0}, {4, 2, 2, 0}, {4, 2, 1, 1}, {4, 2, 1, 0}, {3, 3, 0, 0},
       {3, 2, 1, 0}, {3, 2, 0, 0}, {3, 1, 1, 1}, {3, 1, 1, 0}, {2, 2, 0, 0},
       {2, 1, 1, 0}, {2, 1, 0, 0}, {1, 1, 0, 0}, {1, 0, 0, 0}, {0, 0, 0, 0}}]];]
```

Out[214]=

```
{57.8804, Null}
```

In[215]:=

```
(* Recurrence of the right hand side *)
summandRHS = (-1)^r q^(1/2 r (7+9 r)) qBinomial[2 n, 7/4 (-1 + (-1)^r) + n - 9 r/2, q];
```

In[216]:=

```
AbsoluteTiming[
 recRHSeven = qSR[qFR[summandRHS /. r → 2 r /. (-1) ^ (2 r) → 1, {n}, {r}, {2}, {2}]];]
```

Out[216]=

```
{33.4824, Null}
```

In[217]:=

```
AbsoluteTiming[recRHSodd =
   qSR[qFR[summandRHS /. r → 2 r + 1 /. (-1) ^ (2 r + 1) → -1, {n}, {r}, {2}, {2}]];]
```

Out[217]=

```
{49.9652, Null}
```

In[218]:=

```
recRHSeven == recRHSodd
```

Out[218]=

```
True
```

In[219]:=

```
(* Find the recurrence satisfied by both sides*)
qREGCD[recRHSeven[[1, 1]], recLHS[[1, 1]], SUM[n]]
```

Out[219]=

$$q^{5+4n}(-1+q^{1+n})(1+q^{1+n})(-1+q^{2+n})(1+q^{2+n})(-1+q^{1+2n})(-1+q^{3+2n})\,\mathrm{SUM}[n] - q^{6+4n}(1+q+q^2)(-1+q^{2+n})(1+q^{2+n})(-1+q^{3+2n})(-1+q^{2+2n}+q^{5+2n})\,\mathrm{SUM}[1+n] + q^{7+4n}(1+q+2q^2+q^3+q^4-q^{3+2n}-2q^{4+2n}-3q^{5+2n}-4q^{6+2n}-4q^{7+2n}-3q^{8+2n}-2q^{9+2n}-q^{10+2n}+q^{7+4n}+q^{8+4n}+2q^{9+4n}+2q^{10+4n}+3q^{11+4n}+2q^{12+4n}+2q^{13+4n}+q^{14+4n}+q^{15+4n})\,\mathrm{SUM}[2+n] - q^{8+4n}(1+q^2)(1+q+q^2+q^3+q^4)(-1+q^{4+2n}+q^{7+2n})\,\mathrm{SUM}[3+n] + q^{9+4n}(1-q+q^2)(1+q+q^2)(1+q+q^2+q^3+q^4)\,\mathrm{SUM}[4+n] + (-1-q^{5+2n}-q^{6+2n}-q^{7+2n}-q^{8+2n}-q^{9+2n}-q^{10+2n})\,\mathrm{SUM}[5+n] + \mathrm{SUM}[6+n]$$

In[220]:=

```
(* Initial conditions are checked under
 "Multisums and Bailey's Theorem Applications" section above. *)
```

Eqn (1.5) with k=4 and i=2

In[221]:=

```
(*Recurrence of the left hand side *)
summandLHS = q^(n[1]^2+n[2]+n[2]^2+n[3]+n[3]^2) qBinomial[+2 n - n[1] - n[2], n[1] - n[2], q] ×
    qBinomial[-2 + 2 n - 2 n[1] - n[2] - n[3], n[2] - n[3], q] × qBinomial[
     -4 + 2 n + n[3] - 2 (n[1] + n[2] + n[3]), n[3], q] /. {n[1] → n1, n[2] → n2, n[3] → n3};
```

In[222]:=

```
(* Running

 AbsoluteTiming[recLHS= qSR[qFR[summandLHS,{n},{n1,n2,n3},{2},{2,1,0}]]]
 takes about 8 hours on a mid level personal laptop.

  We instead use the precalculated structure set (see Riese's qMultiSum) here.

 *)
AbsoluteTiming[
 recLHS = qSR[qFR[summandLHS, {n}, {n1, n2, n3}, {0}, {0, 0, 0}, StructSet →
      {{8, 8, 0, 0}, {8, 7, 1, 0}, {8, 6, 2, 0}, {8, 5, 3, 0}, {8, 4, 4, 0}, {7, 7, 0, 0},
       {7, 6, 1, 0}, {7, 6, 0, 0}, {7, 5, 2, 0}, {7, 5, 1, 1}, {7, 5, 1, 0}, {7, 4, 3, 0},
       {7, 4, 2, 1}, {7, 4, 2, 0}, {7, 3, 3, 1}, {6, 6, 0, 0}, {6, 5, 1, 0}, {6, 5, 0, 0},
       {6, 4, 2, 0}, {6, 4, 1, 1}, {6, 4, 1, 0}, {6, 3, 3, 0}, {6, 3, 2, 1}, {6, 3, 2, 0},
       {6, 2, 2, 2}, {5, 5, 0, 0}, {5, 4, 1, 0}, {5, 4, 0, 0}, {5, 3, 2, 0}, {5, 3, 1, 1},
       {5, 3, 1, 0}, {5, 2, 2, 1}, {5, 2, 2, 0}, {4, 4, 0, 0}, {4, 3, 1, 0},
       {4, 3, 0, 0}, {4, 2, 2, 0}, {4, 2, 1, 1}, {4, 2, 1, 0}, {3, 3, 0, 0},
       {3, 2, 1, 0}, {3, 2, 0, 0}, {3, 1, 1, 1}, {3, 1, 1, 0}, {2, 2, 0, 0},
       {2, 1, 1, 0}, {2, 1, 0, 0}, {1, 1, 0, 0}, {1, 0, 0, 0}, {0, 0, 0, 0}}]];]
```

Out[222]=

```
{57.5152, Null}
```

In[223]:=

```
(* Recurrence of the right hand side *)
```

$$\mathrm{summandRHS} = (-1)^r q^{\frac{1}{2} r (5+9r)}\,\mathrm{qBinomial}\left[2n, \frac{5}{4}\left(-1+(-1)^r\right)+n-\frac{9r}{2}, q\right];$$

In[224]:=

```
AbsoluteTiming[
 recRHSeven = qSR[qFR[summandRHS /. r → 2 r /. (-1) ^ (2 r) → 1, {n}, {r}, {2}, {2}]];]
```

Out[224]=

```
{35.5177, Null}
```

In[225]:=

```
AbsoluteTiming[recRHSodd =
   qSR[qFR[summandRHS /. r → 2 r + 1 /. (-1) ^ (2 r + 1) → -1, {n}, {r}, {2}, {2}]];]
```

Out[225]=

```
{36.4644, Null}
```

In[226]:=

```
recRHSeven == recRHSodd
```

Out[226]=

```
True
```

In[227]:=

```
(* Find the recurrence satisfied by both sides*)
qREGCD[recRHSeven[[1, 1]], recLHS[[1, 1]], SUM[n]]
```

Out[227]=

$$q^{7+4n}\left(-1+q^{1+n}\right)\left(1+q^{1+n}\right)\left(-1+q^{2+n}\right)\left(1+q^{2+n}\right)\left(-1+q^{3+2n}\right)\left(-1+q^{5+2n}\right)\text{SUM}[n]-$$
$$q^{8+4n}\left(1+q+q^2\right)\left(-1+q^{2+n}\right)\left(1+q^{2+n}\right)\left(-1+q^{5+2n}\right)\left(-1+q^{3+2n}+q^{6+2n}\right)\text{SUM}[1+n]+$$
$$q^{9+4n}\left(1+q+2q^2+q^3+q^4-q^{4+2n}-2q^{5+2n}-3q^{6+2n}-4q^{7+2n}-4q^{8+2n}-3q^{9+2n}-2q^{10+2n}-q^{11+2n}+\right.$$
$$\left.q^{9+4n}+q^{10+4n}+2q^{11+4n}+2q^{12+4n}+3q^{13+4n}+2q^{14+4n}+2q^{15+4n}+q^{16+4n}+q^{17+4n}\right)\text{SUM}[2+n]-$$
$$q^{10+4n}\left(1+q^2\right)\left(1+q+q^2+q^3+q^4\right)\left(-1+q^{5+2n}+q^{8+2n}\right)\text{SUM}[3+n]+$$
$$q^{11+4n}\left(1-q+q^2\right)\left(1+q+q^2\right)\left(1+q+q^2+q^3+q^4\right)\text{SUM}[4+n]+$$
$$\left(-1-q^{6+2n}-q^{7+2n}-q^{8+2n}-q^{9+2n}-q^{10+2n}-q^{11+2n}\right)\text{SUM}[5+n]+\text{SUM}[6+n]$$

In[228]:=

```
(* Initial conditions are checked under
 "Multisums and Bailey's Theorem Applications" section above. *)
```

Eqn (1.5) with k=4 and i=3

In[229]:=

```
(*Recurrence of the left hand side *)
summandLHS = q^(n[1]^2+n[2]^2+n[3]+n[3]^2) qBinomial[+2 n - n[1] - n[2], n[1] - n[2], q] ×
    qBinomial[+2 n - 2 n[1] - n[2] - n[3], n[2] - n[3], q] × qBinomial[
     -2 + 2 n + n[3] - 2 (n[1] + n[2] + n[3]), n[3], q] /. {n[1] → n1, n[2] → n2, n[3] → n3};
```

In[230]:=

```
(* Running

 AbsoluteTiming[recLHS= qSR[qFR[summandLHS,{n},{n1,n2,n3},{2},{2,1,0}]]]
 takes about 8 hours on a mid level personal laptop.

  We instead use the precalculated structure set (see Riese's qMultiSum) here.

 *)
AbsoluteTiming[
 recLHS = qSR[qFR[summandLHS, {n}, {n1, n2, n3}, {0}, {0, 0, 0}, StructSet →
      {{8, 8, 0, 0}, {8, 7, 1, 0}, {8, 6, 2, 0}, {8, 5, 3, 0}, {8, 4, 4, 0}, {7, 7, 0, 0},
       {7, 6, 1, 0}, {7, 6, 0, 0}, {7, 5, 2, 0}, {7, 5, 1, 1}, {7, 5, 1, 0}, {7, 4, 3, 0},
       {7, 4, 2, 1}, {7, 4, 2, 0}, {7, 3, 3, 1}, {6, 6, 0, 0}, {6, 5, 1, 0}, {6, 5, 0, 0},
       {6, 4, 2, 0}, {6, 4, 1, 1}, {6, 4, 1, 0}, {6, 3, 3, 0}, {6, 3, 2, 1}, {6, 3, 2, 0},
       {6, 2, 2, 2}, {5, 5, 0, 0}, {5, 4, 1, 0}, {5, 4, 0, 0}, {5, 3, 2, 0}, {5, 3, 1, 1},
       {5, 3, 1, 0}, {5, 2, 2, 1}, {5, 2, 2, 0}, {4, 4, 0, 0}, {4, 3, 1, 0},
       {4, 3, 0, 0}, {4, 2, 2, 0}, {4, 2, 1, 1}, {4, 2, 1, 0}, {3, 3, 0, 0},
       {3, 2, 1, 0}, {3, 2, 0, 0}, {3, 1, 1, 1}, {3, 1, 1, 0}, {2, 2, 0, 0},
       {2, 1, 1, 0}, {2, 1, 0, 0}, {1, 1, 0, 0}, {1, 0, 0, 0}, {0, 0, 0, 0}}]];]
```

Out[230]=

{58.7536, Null}

In[231]:=

```
(* Recurrence of the right hand side *)
```

$$\text{summandRHS} = (-1)^r q^{\frac{1}{2} r (3+9 r)} \text{qBinomial}\left[2 n, \frac{3}{4}\left(-1+(-1)^r\right)+n-\frac{9 r}{2}, q\right];$$

In[232]:=

```
AbsoluteTiming[
 recRHSeven = qSR[qFR[summandRHS /. r → 2 r /. (-1) ^ (2 r) → 1, {n}, {r}, {2}, {2}]];]
```

Out[232]=

{34.9148, Null}

In[233]:=

```
AbsoluteTiming[recRHSodd =
   qSR[qFR[summandRHS /. r → 2 r + 1 /. (-1) ^ (2 r + 1) → -1, {n}, {r}, {2}, {2}]];]
```

Out[233]=

{32.1538, Null}

In[234]:=

```
recRHSeven == recRHSodd
```

Out[234]=

True

In[235]:=

```
(* Find the recurrence satisfied by both sides*)
qREGCD[recRHSeven[[1, 1]], recLHS[[1, 1]], SUM[n]]
```

Out[235]=

$$q^{9+4n}\left(-1+q^{1+n}\right)\left(1+q^{1+n}\right)\left(-1+q^{2+n}\right)\left(1+q^{2+n}\right)\left(-1+q^{3+n}\right)\left(1+q^{3+n}\right)\left(-1+q^{1+2n}\right)\left(-1+q^{3+2n}\right)\mathrm{SUM}[n]-q^{10+4n}\left(1+q+q^2\right)\left(-1+q^{2+n}\right)\left(1+q^{2+n}\right)\left(-1+q^{3+n}\right)\left(1+q^{3+n}\right)\left(-1+q^{3+2n}\right)\left(-1+q^{1+2n}+q^{4+2n}\right)\mathrm{SUM}[1+n]+q^{11+4n}\left(-1+q^{3+n}\right)\left(1+q^{3+n}\right)\left(1+q+2q^2+q^3+q^4-q^{1+2n}-q^{2+2n}-2q^{3+2n}-3q^{4+2n}-4q^{5+2n}-4q^{6+2n}-2q^{7+2n}-2q^{8+2n}-q^{9+2n}+q^{5+4n}+q^{6+4n}+2q^{7+4n}+2q^{8+4n}+3q^{9+4n}+2q^{10+4n}+2q^{11+4n}+q^{12+4n}+q^{13+4n}\right)\mathrm{SUM}[2+n]-q^{12+4n}\left(2+q+2q^2+2q^3+2q^4+q^5-q^{1+2n}-q^{2+2n}-2q^{3+2n}-3q^{4+2n}-3q^{5+2n}-5q^{6+2n}-4q^{7+2n}-4q^{8+2n}-3q^{9+2n}-2q^{10+2n}-q^{11+2n}-q^{12+2n}+q^{7+4n}+q^{8+4n}+2q^{9+4n}+3q^{10+4n}+3q^{11+4n}+3q^{12+4n}+3q^{13+4n}+2q^{14+4n}+q^{15+4n}+q^{16+4n}\right)\mathrm{SUM}[3+n]+q^{4+2n}\left(1-q+q^2\right)\left(1+q+q^2\right)\left(-1+q^2-q^{5+2n}-q^{8+2n}-q^{9+2n}-q^{10+2n}-q^{11+2n}+q^{11+4n}+q^{12+4n}+q^{13+4n}+q^{14+4n}+q^{15+4n}\right)\mathrm{SUM}[4+n]+\left(1+q^{7+2n}+q^{8+2n}+q^{9+2n}+q^{10+2n}+q^{11+2n}-q^{10+4n}-q^{11+4n}-q^{12+4n}-q^{13+4n}-q^{14+4n}-q^{15+4n}\right)\mathrm{SUM}[5+n]+\left(-1+q^{2+n}\right)\left(1+q^{2+n}\right)\mathrm{SUM}[6+n]$$

In[236]:=

```
(* Initial conditions are checked under
 "Multisums and Bailey's Theorem Applications" section above. *)
```

Eqn (1.5) with k=4 and i=4

In[237]:=

```
(*Recurrence of the left hand side *)
summandLHS = q^(n[1]^2+n[2]^2+n[3]^2) qBinomial[+2 n - n[1] - n[2], n[1] - n[2], q] ×
    qBinomial[+2 n - 2 n[1] - n[2] - n[3], n[2] - n[3], q] × qBinomial[
     +2 n + n[3] - 2 (n[1] + n[2] + n[3]), n[3], q] /. {n[1] → n1, n[2] → n2, n[3] → n3};
```

In[238]:=

```
(* Running

 AbsoluteTiming[recLHS= qSR[qFR[summandLHS,{n},{n1,n2,n3},{2},{2,1,0}]]]
 takes about 8 hours on a mid level personal laptop.

  We instead use the precalculated structure set (see Riese's qMultiSum) here.

 *)
AbsoluteTiming[
 recLHS = qSR[qFR[summandLHS, {n}, {n1, n2, n3}, {0}, {0, 0, 0}, StructSet →
      {{8, 8, 0, 0}, {8, 7, 1, 0}, {8, 6, 2, 0}, {8, 5, 3, 0}, {8, 4, 4, 0}, {7, 7, 0, 0},
       {7, 6, 1, 0}, {7, 6, 0, 0}, {7, 5, 2, 0}, {7, 5, 1, 1}, {7, 5, 1, 0}, {7, 4, 3, 0},
       {7, 4, 2, 1}, {7, 4, 2, 0}, {7, 3, 3, 1}, {6, 6, 0, 0}, {6, 5, 1, 0}, {6, 5, 0, 0},
       {6, 4, 2, 0}, {6, 4, 1, 1}, {6, 4, 1, 0}, {6, 3, 3, 0}, {6, 3, 2, 1}, {6, 3, 2, 0},
       {6, 2, 2, 2}, {5, 5, 0, 0}, {5, 4, 1, 0}, {5, 4, 0, 0}, {5, 3, 2, 0}, {5, 3, 1, 1},
       {5, 3, 1, 0}, {5, 2, 2, 1}, {5, 2, 2, 0}, {4, 4, 0, 0}, {4, 3, 1, 0},
       {4, 3, 0, 0}, {4, 2, 2, 0}, {4, 2, 1, 1}, {4, 2, 1, 0}, {3, 3, 0, 0},
       {3, 2, 1, 0}, {3, 2, 0, 0}, {3, 1, 1, 1}, {3, 1, 1, 0}, {2, 2, 0, 0},
       {2, 1, 1, 0}, {2, 1, 0, 0}, {1, 1, 0, 0}, {1, 0, 0, 0}, {0, 0, 0, 0}}]];]
```

Out[238]=

```
{58.8618, Null}
```

In[239]:=

```
(* Recurrence of the right hand side *)
summandRHS = (-1)^r q^(1/2 r (1+9 r)) qBinomial[2 n, 1/4 (-1 + (-1)^r) + n - 9 r/2, q];
```

In[240]:=

```
AbsoluteTiming[
 recRHSeven = qSR[qFR[summandRHS /. r → 2 r /. (-1) ^ (2 r) → 1, {n}, {r}, {2}, {2}]];]
```

Out[240]=

```
{35.4382, Null}
```

In[241]:=

```
AbsoluteTiming[recRHSodd =
   qSR[qFR[summandRHS /. r → 2 r + 1 /. (-1) ^ (2 r + 1) → -1, {n}, {r}, {2}, {2}]];]
```

Out[241]=

```
{42.476, Null}
```

In[242]:=

```
recRHSeven == recRHSodd
```

Out[242]=

```
True
```

In[243]:=

```
(* Find the recurrence satisfied by both sides*)
qREGCD[recRHSeven[[1, 1]], recLHS[[1, 1]], SUM[n]]
```

Out[243]=

$$q^{11+4n}\left(-1+q^{1+n}\right)\left(1+q^{1+n}\right)\left(-1+q^{2+n}\right)\left(1+q^{2+n}\right)\left(-1+q^{1+2n}\right)\left(-1+q^{3+2n}\right)\mathrm{SUM}[n]-q^{12+4n}\left(-1+q^{2+n}\right)\left(1+q^{2+n}\right)\left(-1+q^{3+2n}\right)\left(-2-q+q^{1+2n}+q^{2+2n}+q^{3+2n}+q^{4+2n}+q^{5+2n}+q^{6+2n}\right)\mathrm{SUM}[1+n]+q^{13+4n}\left(3+2q+q^2-q^{1+2n}-q^{2+2n}-3q^{3+2n}-4q^{4+2n}-4q^{5+2n}-4q^{6+2n}-2q^{7+2n}-q^{8+2n}+q^{5+4n}+q^{6+4n}+2q^{7+4n}+2q^{8+4n}+3q^{9+4n}+2q^{10+4n}+2q^{11+4n}+q^{12+4n}+q^{13+4n}\right)\mathrm{SUM}[2+n]-q^{7+2n}\left(-1+q^2-q^{5+2n}-q^{6+2n}-3q^{7+2n}-2q^{8+2n}-2q^{9+2n}-q^{10+2n}+q^{8+4n}+q^{9+4n}+2q^{10+4n}+3q^{11+4n}+3q^{12+4n}+3q^{13+4n}+3q^{14+4n}+2q^{15+4n}+q^{16+4n}+q^{17+4n}\right)\mathrm{SUM}[3+n]+q^{6+2n}\left(1-q^4+q^{5+2n}+q^{6+2n}+2q^{7+2n}+2q^{8+2n}+3q^{9+2n}+2q^{10+2n}+2q^{11+2n}+q^{12+2n}+q^{13+2n}\right)\mathrm{SUM}[4+n]+\left(-1-q^{6+2n}-q^{7+2n}-q^{8+2n}-q^{9+2n}-q^{10+2n}-q^{11+2n}\right)\mathrm{SUM}[5+n]+\mathrm{SUM}[6+n]$$

In[244]:=

```
(* Initial conditions are checked under
 "Multisums and Bailey's Theorem Applications" section above. *)
```

Proofs of Theorem 1.7 with k=4

Eqn (1.8) with k=4 and i=1

In[245]:=

```
(*Recurrence of the left hand side *)
summandLHS = q^(n[1]+n[1]^2+n[2]+n[2]^2+n[3]+n[3]^2) qBinomial[-3 + n - n[1] - n[2], n[3], q^2] ×
    qBinomial[-2 + 2 n - n[1] - n[2], n[1] - n[2], q] × qBinomial[
     -4 + 2 n - 2 n[1] - n[2] - n[3], n[2] - n[3], q] /. {n[1] → n1, n[2] → n2, n[3] → n3};
```

In[246]:=

```
(* Running

 AbsoluteTiming[recLHS= qSR[qFR[summandLHS,{n},{n1,n2,n3},{2},{2,1,0}]]]
 takes about an hour on a mid level personal laptop.

  We instead use the precalculated structure set (see Riese's qMultiSum) here.

 *)
AbsoluteTiming[
 recLHS = qSR[qFR[summandLHS, {n}, {n1, n2, n3}, {0}, {0, 0, 0}, StructSet →
      {{4, 4, 1, 0}, {4, 3, 2, 0}, {4, 2, 3, 0}, {3, 3, 1, 0}, {3, 2, 2, 0}, {3, 2, 1, 0},
       {3, 1, 2, 1}, {2, 2, 1, 0}, {2, 1, 2, 0}, {2, 1, 1, 0},
       {1, 1, 1, 0}, {1, 0, 1, 0}, {0, 0, 1, 0}}]];]
```

Out[246]=

```
{0.605284, Null}
```

In[247]:=

```
(* Recurrence of the right hand side *)
```

summandRHS = $(-1)^r q^{r(3+4r)}$ qBinomial$\left[2n, \frac{3}{2}\left(-1+(-1)^r\right)+n-4r, q\right]$;

In[248]:=

```
AbsoluteTiming[
 recRHSeven = qSR[qFR[summandRHS /. r → 2 r /. (-1) ^ (2 r) → 1, {n}, {r}, {2}, {2}]];]
```

Out[248]=

```
{28.9329, Null}
```

In[249]:=

```
AbsoluteTiming[recRHSodd =
   qSR[qFR[summandRHS /. r → 2 r + 1 /. (-1) ^ (2 r + 1) → -1, {n}, {r}, {2}, {2}]];]
```

Out[249]=

```
{28.7617, Null}
```

In[250]:=

```
recRHSeven == recRHSodd
```

Out[250]=

```
True
```

In[251]:=

```
(* Find the recurrence satisfied by both sides*)
qREGCD[recRHSeven⟦1, 1⟧, recLHS⟦1, 1⟧, SUM[n]]
```

Out[251]=

$$-q^{2n}\left(-1+q^{1+n}\right)\left(1+q^{1+n}\right)\left(-1+q^{1+2n}\right)\mathrm{SUM}[n]+ q^{1+2n}\left(-1+q^{2+2n}+q^{3+2n}+q^{4+2n}\right)\mathrm{SUM}[1+n]+\left(-1-q^{2+2n}-q^{3+2n}-q^{4+2n}\right)\mathrm{SUM}[2+n]+\mathrm{SUM}[3+n]$$

In[252]:=

```
(* Initial conditions are checked under
 "Multisums and Bailey's Theorem Applications" section above. *)
```

Eqn (1.8) with k=4 and i=2

In[253]:=

```
(*Recurrence of the left hand side *)
summandLHS = q^(n[1]^2+n[2]+n[2]^2+n[3]+n[3]^2) qBinomial[-2 + n - n[1] - n[2], n[3], q^2] ×
    qBinomial[+2 n - n[1] - n[2], n[1] - n[2], q] × qBinomial[
     -2 + 2 n - 2 n[1] - n[2] - n[3], n[2] - n[3], q] /. {n[1] → n1, n[2] → n2, n[3] → n3};
```

In[254]:=

```
(* Running

 AbsoluteTiming[recLHS= qSR[qFR[summandLHS,{n},{n1,n2,n3},{2},{2,1,0}]]]
 takes about an hour on a mid level personal laptop.

  We instead use the precalculated structure set (see Riese's qMultiSum) here.

 *)
AbsoluteTiming[
 recLHS = qSR[qFR[summandLHS, {n}, {n1, n2, n3}, {0}, {0, 0, 0}, StructSet →
      {{4, 4, 1, 0}, {4, 3, 2, 0}, {4, 2, 3, 0}, {3, 3, 1, 0}, {3, 2, 2, 0}, {3, 2, 1, 0},
       {3, 1, 2, 1}, {2, 2, 1, 0}, {2, 1, 2, 0}, {2, 1, 1, 0},
       {1, 1, 1, 0}, {1, 0, 1, 0}, {0, 0, 1, 0}}]];]
```

Out[254]=

```
{0.554447, Null}
```

In[255]:=

```
(* Recurrence of the right hand side *)
summandRHS = (-1)^r q^(r (2+4 r)) qBinomial[2 n, 2/2 (-1 + (-1)^r) + n - 4 r, q];
```

In[256]:=

```
AbsoluteTiming[
 recRHSeven = qSR[qFR[summandRHS /. r → 2 r /. (-1) ^ (2 r) → 1, {n}, {r}, {2}, {2}]];]
```

Out[256]=

```
{26.8635, Null}
```

In[257]:=

```
AbsoluteTiming[recRHSodd =
   qSR[qFR[summandRHS /. r → 2 r + 1 /. (-1) ^ (2 r + 1) → -1, {n}, {r}, {2}, {2}]];]
```

Out[257]=

```
{25.1841, Null}
```

In[258]:=

```
recRHSeven == recRHSodd
```

Out[258]=

```
True
```

In[259]:=

```
(* Find the recurrence satisfied by both sides*)
qREGCD[recRHSeven[[1, 1]], recLHS[[1, 1]], SUM[n]]
```

Out[259]=

$$-q^{1+2n}\left(-1+q^{1+n}\right)\left(1+q^{1+n}\right)\left(-1+q^{3+2n}\right)\mathrm{SUM}[n]+ q^{2+2n}\left(-1+q^{3+2n}+q^{4+2n}+q^{5+2n}\right)\mathrm{SUM}[1+n]+\left(-1-q^{3+2n}-q^{4+2n}-q^{5+2n}\right)\mathrm{SUM}[2+n]+\mathrm{SUM}[3+n]$$

In[260]:=
```
(* Initial conditions are checked under
 "Multisums and Bailey's Theorem Applications" section above. *)
```

Eqn (1.8) with k=4 and i=3

In[261]:=
```
(*Recurrence of the left hand side *)
summandLHS = q^(n[1]^2+n[2]^2+n[3]+n[3]^2) qBinomial[-1 + n - n[1] - n[2], n[3], q^2] ×
    qBinomial[+2 n - n[1] - n[2], n[1] - n[2], q] × qBinomial[
     +2 n - 2 n[1] - n[2] - n[3], n[2] - n[3], q] /. {n[1] → n1, n[2] → n2, n[3] → n3};
```

In[262]:=
```
(* Running

 AbsoluteTiming[recLHS= qSR[qFR[summandLHS,{n},{n1,n2,n3},{2},{2,1,0}]]]
 takes about an hour on a mid level personal laptop.

  We instead use the precalculated structure set (see Riese's qMultiSum) here.

 *)
AbsoluteTiming[
 recLHS = qSR[qFR[summandLHS, {n}, {n1, n2, n3}, {0}, {0, 0, 0}, StructSet →
      {{4, 4, 1, 0}, {4, 3, 2, 0}, {4, 2, 3, 0}, {3, 3, 1, 0}, {3, 2, 2, 0}, {3, 2, 1, 0},
       {3, 1, 2, 1}, {2, 2, 1, 0}, {2, 1, 2, 0}, {2, 1, 1, 0},
       {1, 1, 1, 0}, {1, 0, 1, 0}, {0, 0, 1, 0}}]];]
```

Out[262]=
```
{0.416854, Null}
```

In[263]:=
```
(* Recurrence of the right hand side *)
summandRHS = (-1)^r q^(r (1+4 r)) qBinomial[2 n, 1/2 (-1 + (-1)^r) + n - 4 r, q];
```

In[264]:=
```
AbsoluteTiming[
 recRHSeven = qSR[qFR[summandRHS /. r → 2 r /. (-1) ^ (2 r) → 1, {n}, {r}, {2}, {2}]];]
```

Out[264]=
```
{19.1621, Null}
```

In[265]:=
```
AbsoluteTiming[recRHSodd =
   qSR[qFR[summandRHS /. r → 2 r + 1 /. (-1) ^ (2 r + 1) → -1, {n}, {r}, {2}, {2}]];]
```

Out[265]=
```
{27.8032, Null}
```

In[266]:=
```
recRHSeven == recRHSodd
```

Out[266]=
```
True
```

In[267]:=

```
(* Find the recurrence satisfied by both sides*)
qREGCD[recRHSeven[[1, 1]], recLHS[[1, 1]], SUM[n]]
```

Out[267]=

$$-q^{2+2n}\left(-1+q^{1+n}\right)\left(1+q^{1+n}\right)\left(-1+q^{1+2n}\right)\text{SUM}[n]+q^{2+2n}\left(1-q-q^{2}+q^{2+2n}+q^{3+2n}+q^{5+2n}\right)\text{SUM}[1+n]+\left(-1-q^{2+2n}-q^{4+2n}-q^{5+2n}\right)\text{SUM}[2+n]+\text{SUM}[3+n]$$

In[268]:=

```
(* Initial conditions are checked under
 "Multisums and Bailey's Theorem Applications" section above. *)
```

Eqn (1.8) with k=4 and i=4

In[269]:=

```
(*Recurrence of the left hand side *)
summandLHS = q^(n[1]^2+n[2]^2+n[3]^2) qBinomial[+n - n[1] - n[2], n[3], q^2] ×
    qBinomial[+2 n - n[1] - n[2], n[1] - n[2], q] × qBinomial[
     +2 n - 2 n[1] - n[2] - n[3], n[2] - n[3], q] /. {n[1] → n1, n[2] → n2, n[3] → n3};
```

In[270]:=

```
(* Running

 AbsoluteTiming[recLHS= qSR[qFR[summandLHS,{n},{n1,n2,n3},{2},{2,1,0}]]]
 takes about an hour on a mid level personal laptop.

  We instead use the precalculated structure set (see Riese's qMultiSum) here.

 *)
AbsoluteTiming[
 recLHS = qSR[qFR[summandLHS, {n}, {n1, n2, n3}, {0}, {0, 0, 0}, StructSet →
      {{4, 4, 1, 0}, {4, 3, 2, 0}, {4, 2, 3, 0}, {3, 3, 1, 0}, {3, 2, 2, 0}, {3, 2, 1, 0},
       {3, 1, 2, 1}, {2, 2, 1, 0}, {2, 1, 2, 0}, {2, 1, 1, 0},
       {1, 1, 1, 0}, {1, 0, 1, 0}, {0, 0, 1, 0}}]];]
```

Out[270]=

```
{0.290233, Null}
```

In[271]:=

```
(* Recurrence of the right hand side *)
summandRHS = (-1)^r q^(r (4 r)) qBinomial[2 n, n - 4 r, q];
```

In[272]:=

```
AbsoluteTiming[
 recRHSeven = qSR[qFR[summandRHS /. r → 2 r /. (-1) ^ (2 r) → 1, {n}, {r}, {2}, {2}]];]
```

Out[272]=

```
{19.2346, Null}
```

In[273]:=

```
AbsoluteTiming[recRHSodd =
   qSR[qFR[summandRHS /. r → 2 r + 1 /. (-1) ^ (2 r + 1) → -1, {n}, {r}, {2}, {2}]];]
```

Out[273]=

```
{18.7121, Null}
```

In[274]:=

```
recRHSeven == recRHSodd
```

Out[274]=

```
True
```

In[275]:=

```
(* Find the recurrence satisfied by both sides*)
qREGCD[recRHSeven[[1, 1]], recLHS[[1, 1]], SUM[n]]
```

Out[275]=

$$-q^{3+2n}\left(-1+q^{1+n}\right)\left(1+q^{1+n}\right)\left(-1+q^{1+2n}\right)\mathrm{SUM}[n]+ q^{4+2n}\left(-1+q^{1+2n}+q^{2+2n}+q^{3+2n}\right)\mathrm{SUM}[1+n]+\left(-1-q^{3+2n}-q^{4+2n}-q^{5+2n}\right)\mathrm{SUM}[2+n]+\mathrm{SUM}[3+n]$$

In[276]:=

```
(* Initial conditions are checked under
 "Multisums and Bailey's Theorem Applications" section above. *)
```
